# Healthcare and Psychosocial Needs in Achondroplasia Across the Lifespan: Developmental Functioning, Multidisciplinary Care, and Family-Centered Outcomes

**DOI:** 10.3390/healthcare14162623

**Published:** 2026-08-19

**Authors:** Rebecca Cristiana Șerban, Andreea Mitut-Veliscu, Alexandra Dumitra, Liana Marica, Cristina Popescu, Andrei Costache, Șerban Teona, Anca-Lelia Riza, Rodica Dirnu, Ion Dorin Pluta, Renata-Maria Varut, Ioana Streata

**Affiliations:** 1 Regional Centre of Medical Genetics Dolj, Emergency County Hospital Craiova, 200642 Craiova, Romania; rebecca.serban@umfcv.ro (R.C.Ș.); alexandra.dumitra@umfcv.ro (A.D.); lianamaricamarica@gmail.com (L.M.); teona.tanciu@gmail.com (Ș.T.); ioana.streata@umfcv.ro (I.S.); 2 Laboratory of Human Genomics, University of Medicine and Pharmacy of Craiova, 200638 Craiova, Romaniaanca.riza@umfcv.ro (A.-L.R.); 3 Doctoral School, University of Medicine and Pharmacy of Craiova, 200349 Craiova, Romania; 4 ENT County Hospital Craiova, Discipline of Anatomy, Department of Anatomy, University of Medicine and Pharmacy, 200349 Craiova, Romania; 5 Department of Biophysics, University of Medicine and Pharmacy of Craiova, 200638 Craiova, Romania; 6 Department of Health and Motricity, Faculty of Medical and Behavioral Sciences, Constantin Brâncuși University of Târgu Jiu, 210185 Târgu Jiu, Romania; rodica.dirnu@e-ucb.ro (R.D.); dorin.pluta@e-ucb.ro (I.D.P.); 7 Research Methodology Department, Faculty of Pharmacy, University of Medicine and Pharmacy of Craiova, 200349 Craiova, Romania; renata.varut@umfcv.ro

**Keywords:** achondroplasia, genetic syndrome, developmental functioning, adaptive functioning, healthcare services, multidisciplinary care, transition to adulthood, quality of life, caregiver burden, family-centered care

## Abstract

Background/Objectives: Achondroplasia is the most common skeletal dysplasia and the leading genetic cause of disproportionate short stature. Although its biological basis involves gain-of-function variants in the FGFR3 gene, achondroplasia is a lifelong multisystem disorder associated with neurological, respiratory, orthopedic, otolaryngological, cardiovascular, oral, functional, and psychosocial complications. This narrative review aims to synthesize the evidence on developmental and adaptive functioning, age-specific healthcare needs, multidisciplinary service delivery, transition to adult care, psychosocial well-being, caregiver burden, and patient- and family-centered outcomes in achondroplasia across the lifespan. Methods: A narrative literature review was conducted using PubMed/MEDLINE, Scopus, Web of Science Core Collection, and CINAHL, with Google Scholar used as a supplementary source. Studies published between January 2010 and July 2026 were considered, together with earlier clinically relevant reports. Evidence addressing prenatal and postnatal diagnosis, age-specific manifestations, neurological and respiratory complications, orthopedic and otolaryngological care, cardiometabolic risk, growth monitoring, multidisciplinary management, transition to adult services, disease-modifying therapy, quality of life, and caregiver burden was evaluated. Results: The clinical priorities of achondroplasia change substantially across the lifespan. Infancy is characterized by an increased risk of foramen magnum stenosis, cervicomedullary compression, hypotonia, and sleep-disordered breathing, whereas orthopedic deformities, chronic pain, reduced mobility, spinal stenosis, hearing impairment, obesity, and cardiovascular risk become increasingly relevant during later childhood, adolescence, and adulthood. Early diagnosis, condition-specific imaging, neurological and respiratory surveillance, growth monitoring, and coordinated specialist care are essential for preventing severe complications. Vosoritide has introduced a disease-modifying therapeutic option, but it does not replace comprehensive clinical surveillance, rehabilitation, orthopedic care, psychosocial support, or shared decision-making. Functional limitations, environmental barriers, treatment burden, and caregiver stress contribute substantially to reduced quality of life. Conclusions: Achondroplasia should be managed as a lifelong multisystem condition rather than solely as a disorder of short stature. Standardized surveillance, multidisciplinary coordination, planned transition to adult care, and patient- and family-centered management are essential for improving function, autonomy, long-term health outcomes, and quality of life.

## 1. Introduction

Achondroplasia is the most common form of disproportionate short stature caused by a skeletal dysplasia and represents a lifelong multisystem condition with clinical consequences extending far beyond impaired linear growth. Its estimated prevalence varies across populations and studies, ranging from approximately 1 in 10,000 to 1 in 30,000 live births, with other estimates reporting 3.72–4.6 cases per 100,000 births [1,2,3]. Despite its classification as a rare disorder, achondroplasia has substantial clinical relevance because affected individuals may develop age-dependent neurological, respiratory, orthopedic, otolaryngological, cardiovascular, functional, and psychosocial complications requiring coordinated long-term care.

The disorder is caused by a heterozygous gain-of-function variant in the *FGFR3* gene, most commonly the p.Gly380Arg substitution. Constitutive activation of fibroblast growth factor receptor 3 impairs chondrocyte proliferation and differentiation and disrupts endochondral ossification, resulting in reduced longitudinal bone growth. Because endochondral ossification is also essential for the development of the skull base, vertebral column, and other skeletal structures, excessive FGFR3 signaling contributes to a broad clinical phenotype rather than to short stature alone [4,5,6,7,8].

The diagnosis is frequently suspected prenatally, at birth, or during early infancy because of the characteristic phenotype. Typical findings include rhizomelic shortening of the limbs, relative macrocephaly with frontal bossing, midface hypoplasia, brachydactyly with a trident hand configuration, and progressive musculoskeletal abnormalities such as lower-limb bowing and spinal deformities [9,10]. Cognitive development is generally normal; however, affected individuals may develop complications involving the craniovertebral junction, spine, respiratory tract, otolaryngological system, cardiovascular system, oral cavity, and locomotor apparatus. These include foramen magnum stenosis, cervicomedullary compression, spinal stenosis, sleep-disordered breathing, recurrent otitis media, hearing impairment, chronic pain, and lower-limb malalignment [11,12,13,14,15].

The clinical priorities of achondroplasia change substantially across the lifespan. During infancy and early childhood, the principal concerns include foramen magnum stenosis, neurological compromise, hypotonia, delayed motor development, and obstructive or central sleep apnea. During school age and adolescence, orthopedic deformities, chronic pain, reduced exercise tolerance, hearing and speech difficulties, and increasing limitations in physical independence may become more prominent. In adulthood, spinal stenosis, degenerative musculoskeletal disease, persistent pain, reduced mobility, obesity, cardiovascular risk, and psychosocial difficulties may contribute substantially to morbidity and reduced well-being [4,9,14].

The impact of achondroplasia also extends to daily functioning, autonomy, psychological health, and social participation. Many individuals live in environments designed for people of average stature, which may create barriers to self-care, education, employment, transportation, healthcare access, and participation in social or recreational activities. Chronic pain, fatigue, restricted mobility, stigma, unwanted attention, and difficulties related to body image or social inclusion may further reduce quality of life. Clinical outcomes should therefore not be evaluated solely in terms of height or skeletal abnormalities, but should also include function, independence, participation, emotional well-being, and patient-reported outcomes.

Families and caregivers are similarly affected by the lifelong nature of the condition. They may need to coordinate repeated medical appointments, specialist consultations, diagnostic investigations, rehabilitation, surgical procedures, and complex therapeutic decisions. These responsibilities can generate emotional, organizational, social, and financial strain. Differences may also exist between how children and their parents perceive the severity and impact of achondroplasia, emphasizing the importance of evaluating both patient and caregiver perspectives. Family education, psychological support, shared decision-making, and access to patient organizations should therefore be considered integral components of care [1,4,14].

Continuity of care remains a major challenge. Pediatric follow-up is increasingly organized in specialized multidisciplinary centers, whereas adult services may be fragmented or less familiar with achondroplasia-specific complications. Some individuals are lost during the transition from pediatric to adult care, despite the persistence or progression of neurological, orthopedic, respiratory, cardiovascular, and functional problems. Transition should therefore be planned as a gradual clinical process that includes patient education, transfer of responsibility, recognition of warning symptoms, and establishment of clear referral pathways. Lifelong multidisciplinary surveillance is essential because the risk of complications does not disappear after childhood but changes in pattern, severity, and clinical relevance [9,13].

The therapeutic landscape has also evolved. Traditional management has relied on clinical surveillance, rehabilitation, environmental adaptation, and medical or surgical treatment of complications. The introduction of vosoritide, the first disease-modifying pharmacological therapy for achondroplasia, has expanded treatment beyond supportive care. Nevertheless, growth-promoting therapy does not replace neurological, respiratory, orthopedic, otolaryngological, cardiovascular, dental, or psychosocial monitoring. Instead, it should be integrated into a comprehensive care pathway that considers age, residual growth potential, complication profile, functional needs, treatment burden, and the preferences of patients and families [15,16,17,18,19,20,21,22,23,24].

In view of the multisystem nature of achondroplasia and the changing clinical priorities across the lifespan, an updated clinically oriented synthesis of the available evidence is needed. Although several comprehensive reviews, consensus statements, and clinical guidelines have addressed the diagnosis, complications, and management of achondroplasia, important gaps remain in the integration of these domains across the lifespan. Evidence concerning developmental and adaptive functioning, transition from pediatric to adult healthcare, psychosocial burden, caregiver experience, environmental barriers, and the integration of disease-modifying therapy into multidisciplinary care is often reported separately, making translation into a unified long-term care pathway difficult. Moreover, several areas of care remain supported predominantly by expert consensus rather than longitudinal empirical evidence. Therefore, the primary aim of this narrative review is to provide a lifespan-oriented clinical framework integrating age-specific healthcare needs with developmental and functional outcomes, multidisciplinary care, transition planning, psychosocial well-being, and family-centered management. A secondary aim is to identify areas supported by robust empirical evidence and those in which clinically important uncertainties remain.

## 2. Materials and Methods

### 2.1. Literature Search Strategy

This narrative review was conducted to provide an updated, clinically oriented synthesis of achondroplasia across the lifespan. The literature search focused on age-specific clinical manifestations, major complications, diagnostic and surveillance strategies, multidisciplinary management, transition from pediatric to adult care, disease-modifying treatment in clinical practice, quality of life, psychosocial impact, and patient- and family-centered outcomes.

A comprehensive literature search was performed in PubMed/MEDLINE, Scopus, Web of Science Core Collection, and CINAHL. Google Scholar was used as a supplementary source for citation tracking and identification of additional relevant publications. The electronic search was complemented by manual screening of the reference lists of relevant original studies, reviews, clinical guidelines, and consensus statements. Articles published between January 2010 and July 2026 were considered, although earlier publications were included when they provided essential information regarding the natural history, diagnosis, or established management of achondroplasia.

### 2.2. Eligibility and Study Selection

The search strategy included combinations of the following terms: “achondroplasia”, “clinical manifestations”, “lifelong care”, “prenatal diagnosis”, “foramen magnum stenosis”, “cervicomedullary compression”, “spinal stenosis”, “sleep-disordered breathing”, “obstructive sleep apnea”, “otitis media”, “hearing impairment”, “orthopedic complications”, “lower-limb deformity”, “chronic pain”, “obesity”, “cardiovascular risk”, “oral manifestations”, “growth monitoring”, “multidisciplinary management”, “vosoritide”, “transition to adult care”, “quality of life”, “psychosocial impact”, “caregiver burden”, “patient-reported outcomes”, and “shared decision-making”.

Original clinical studies, observational studies, clinical trials, consensus statements, professional recommendations, and relevant reviews published in English were considered. Both pediatric and adult studies were evaluated to reflect the changing clinical priorities and cumulative burden of achondroplasia throughout life. Particular emphasis was placed on clinically applicable evidence concerning early recognition of severe complications, age-specific surveillance, coordinated multidisciplinary care, functional outcomes, quality of life, and the integration of disease-modifying treatment into comprehensive management. The literature was selected according to its relevance to the objectives of the review, methodological quality, clinical applicability, and contribution to understanding the lifelong course of achondroplasia. Because this article was designed as a narrative review, no formal systematic-review protocol, risk-of-bias assessment, or quantitative meta-analysis was performed. The selected evidence was critically synthesized into a clinically oriented framework emphasizing prevention of complications, continuity of care, functional independence, and patient- and family-centered outcomes. Titles and abstracts were screened for relevance to the predefined objectives of the review. Potentially relevant publications underwent full-text assessment. Studies were retained when they provided clinically relevant evidence concerning at least one predefined review domain. Duplicate publications, articles without sufficient relevance to achondroplasia care, and publications lacking clinically interpretable information were excluded. Reference lists of key publications were additionally screened to identify relevant studies not retrieved by the electronic search. Following preliminary relevance screening, 66 potentially relevant publications were considered. Of these, 47 underwent full-text assessment, and 31 publications meeting the predefined eligibility and relevance criteria were retained for the final narrative synthesis.

### 2.3. Narrative Synthesis and Content Analysis

The selected literature was analyzed using a structured narrative synthesis informed by directed qualitative content analysis, following established methodological recommendations for narrative reviews and qualitative content analysis [25,26,27,28]. This approach was considered appropriate because the review integrates heterogeneous evidence from observational studies, clinical trials, natural-history studies, consensus statements, clinical guidelines, and psychosocial and health-services research rather than addressing a single intervention suitable for quantitative pooling.

Following study selection, relevant information was extracted into a structured evidence matrix including study design, population and age group, clinical domain, source of evidence, principal findings, and reported limitations. A directed content-analysis approach was then applied using predefined domains derived from the objectives of the review: (1) developmental and adaptive functioning; (2) age-specific clinical manifestations and complications; (3) diagnosis and surveillance; (4) multidisciplinary care; (5) transition from pediatric to adult healthcare; (6) disease-modifying therapy; and (7) psychosocial, caregiver, and family-centered outcomes. Additional recurrent themes were incorporated when relevant [27,28].

Evidence derived from empirical studies was considered separately from recommendations based primarily on clinical guidelines, consensus statements, or expert opinion. Areas of agreement, uncertainty, inconsistency, and limited evidence were identified within each thematic domain. No quantitative meta-analysis was performed.

## 3. Clinical Manifestations

### 3.1. Prenatal and Neonatal Features

Achondroplasia can be difficult to recognize in early pregnancy, as the typical features are often absent during the first and second trimesters. The routine fetal anatomy scan at 18–20 weeks may appear normal, and suspicion is therefore more likely to arise later, when disproportionate skeletal growth becomes easier to detect. This delayed recognition is important clinically, because a normal mid-trimester scan does not exclude achondroplasia when later growth patterns become suggestive.

On prenatal ultrasound, the most useful clue is progressive shortening of the long bones, particularly the femur, usually reflecting the beginning of rhizomelic limb disproportion. The discrepancy between femur length and head size may become increasingly apparent in the third trimester, especially when relative macrocephaly is present. Additional sonographic findings may include frontal bossing, a depressed nasal bridge, brachydactyly with a trident hand configuration, and the so-called collar hoop sign, which reflects abnormal metaphyseal morphology [15]. Shortening of the humerus may also support the suspicion, particularly when it occurs together with femoral shortening and craniofacial features. These ultrasound findings are not specific when considered in isolation. Femoral shortening, for example, may be seen in other skeletal dysplasias or in fetal growth restriction, so interpretation depends on the overall pattern of disproportion, cranial morphology, limb measurements, and family history. In selected cases, polyhydramnios may also be reported, but it is not a defining feature and should be interpreted cautiously. For this reason, prenatal ultrasound is best viewed as a tool that raises suspicion rather than one that establishes the diagnosis by itself. When findings are suggestive, referral for specialist fetal assessment and molecular confirmation should be considered [15,29].

The neonatal period can also be diagnostically variable. Some newborns show clear features from the first examination, including relative macrocephaly, shortening of the proximal limbs, and early craniofacial disproportion. In others, the phenotype is subtler. Body length may still fall within the expected range for neonates of average-stature parents, and midface hypoplasia may be mild in the first days of life, becoming more apparent only later [15]. This variability makes careful observation important in both prenatal and neonatal settings. When achondroplasia is suspected, early postnatal follow-up allows clinical assessment, genetic confirmation, and timely surveillance for complications that are most relevant during infancy.

### 3.2. Craniofacial Characteristics

Craniofacial changes are a central part of the achondroplasia phenotype and often provide early clinical clues. Macrocephaly and frontal bossing are among the most recognizable findings, giving the head a prominent, rounded appearance [5,15]. They may be visible in infancy, although they often become more obvious with growth. Midface hypoplasia is another defining feature. It reflects impaired development of the skull base, which depends partly on endochondral ossification [7,15]. Clinically, this produces a retruded midface and a depressed nasal bridge, contributing to the typical facial profile of achondroplasia. In some patients, cranial shape may also be influenced by partial premature fusion of the coronal or sagittal sutures [7].

The mandible can be involved as well. Experimental and imaging data suggest that altered mandibular cartilage development may lead to mandibular hypoplasia, micrognathia, and abnormal mandibular shape. These changes are not only morphological. They may contribute to feeding difficulties, malocclusion, and upper airway compromise, especially when midface hypoplasia is also present [19]. Findings from animal models help explain the structural basis of these abnormalities. In murine achondroplasia, craniofacial changes include skull base shortening, a dome-shaped skull, maxillo-mandibular misalignment, and premature fusion of cranial synchondroses. Their partial improvement after reduction in FGFR3 expression supports the link between craniofacial development and the degree of receptor overactivity [5]. The craniofacial phenotype is therefore diagnostically useful, but its importance is not limited to recognition of the disorder. Abnormal skull base development, midface retrusion, and mandibular dysmorphology can also influence feeding, breathing, and functional well-being.

### 3.3. Skeletal Abnormalities

Skeletal abnormalities form the core clinical expression of achondroplasia and involve both the axial and appendicular skeleton. The most evident feature is rhizomelic limb shortening, present in nearly all patients and increasingly apparent with growth [7,15]. This disproportion reflects impaired endochondral ossification and gives the disorder its characteristic short-limbed phenotype. Spinal involvement is frequent and clinically important. Lumbar lordosis, thoracolumbar kyphosis, and progressive narrowing of the spinal canal may contribute to postural imbalance, pain, and, in more severe cases, neurological compromise [7,21,24]. Thoracolumbar kyphosis is especially common during infancy and often improves once independent walking develops, although some children retain a persistent deformity [21]. Radiologically, progressive narrowing of the lumbar interpedicular distance is a typical finding and contributes to the anatomical basis of spinal stenosis [15,29].

Lower limb deformities are often complex rather than isolated. Genu varum is one of the most common orthopedic manifestations, but it is frequently associated with internal tibial torsion, recurvatum, and abnormal loading across the knee and ankle [21]. Other features may include bowing of the legs, ligamentous laxity, hip flexion contractures, and ankle malalignment [16]. These abnormalities can disturb gait mechanics and may become painful, particularly in older children, adolescents, and adults [16,21]. For this reason, orthopedic assessment needs to consider the entire limb and gait pattern, not only a single angular deformity. Upper limb involvement is also common, although it receives less attention than lower limb deformity. Typical findings include brachydactyly, short fingers, and the classic trident hand [15,16]. Some patients also have limited elbow extension, reduced pronation-supination, distal humeral bowing, radial head deformity or subluxation, and relative ulnar shortening. These changes may affect reach, self-care, and other daily activities requiring upper limb function [16].

Radiographic features help define the skeletal phenotype and are useful in both diagnosis and follow-up. Common findings include square iliac bones, narrow sacrosciatic notches, metaphyseal flaring, short femoral necks, and narrowing of the lumbar interpedicular distance [15,29]. Together, these features reflect the generalized nature of the skeletal dysplasia and help distinguish achondroplasia from other causes of disproportionate short stature. The disease may also affect bone quality, not only bone size and shape. Experimental and imaging studies have described reduced cortical thickness, altered trabecular architecture, and osteopenia in some contexts [7]. This broader skeletal involvement is consistent with evidence that long bones, vertebrae, and cranial structures are all affected by excessive FGFR3 activity [5]. Skeletal disease in achondroplasia is therefore generalized, progressive, and functionally important. It contributes substantially to chronic pain, gait disturbance, reduced mobility, and the need for repeated orthopedic surveillance or intervention across the lifespan [14,21].

### 3.4. Neurological Features

Neurological manifestations in achondroplasia are usually secondary to skeletal abnormalities, especially those affecting the foramen magnum, cranio-cervical junction, and spinal canal. Narrowing at these sites can compress the brainstem, upper cervical cord, or more distal neural structures, making neurological surveillance one of the most important parts of clinical care [5,15,16]. In infancy, the major concern is foramen magnum stenosis with cervicomedullary compression. This may present with central apnea, generalized hypotonia, delayed motor development, or, in severe cases, life-threatening neurological compromise [12,15]. Hypotonia may already be present in the neonatal period, which justifies careful assessment from the earliest stages of life [14,15].

Neurological evaluation in infants should be systematic. It includes assessment of tone, strength, reflexes, cranial nerve function, feeding, spontaneous movements, and gross motor development [15]. A practical difficulty is that children with achondroplasia do not follow exactly the same developmental trajectory as average-stature peers. Motor milestones should therefore be interpreted using achondroplasia-specific expectations, rather than standard pediatric norms alone [15]. Several findings should prompt urgent investigation for cervicomedullary compression. These include persistent hypotonia, hyperreflexia, sustained ankle clonus, asymmetric reflexes, early hand preference, marked developmental delay, irritability, swallowing difficulties, and rapid head growth beyond achondroplasia-specific curves [12,16]. Their importance lies in the fact that neurological compromise may begin subtly, before becoming clinically obvious. Hydrocephalus is another neurological feature to consider. It is less frequent, but has been reported in clinical cohorts and may contribute to progressive head enlargement and additional neurological risk [5,29]. More broadly, abnormalities of the skull base and vertebral column create a structural setting in which neurological symptoms may appear not only in infancy, but also later in life.

Spinal stenosis beyond the cranio-cervical junction may also produce neurological symptoms. It usually reflects congenital canal narrowing combined with later degenerative changes. Clinically, it may lead to gait disturbance, limb weakness, paraparesis or quadriparesis, myelopathy, and chronic neurological impairment [5,7]. This illustrates how closely neurological manifestations in achondroplasia are linked to the underlying skeletal disorder. The neurological spectrum is therefore broad, ranging from early hypotonia and delayed motor development to serious brainstem or spinal cord compression. Because symptoms may be subtle at first, structured neurological follow-up remains essential throughout life.

### 3.5. Other Systemic Manifestations

Although achondroplasia is usually described as a skeletal dysplasia, its clinical impact extends well beyond the musculoskeletal system. Patients may develop respiratory, ENT, neurological, metabolic, and functional complications, many of which contribute significantly to long-term morbidity [14,15]. Cognitive development is generally normal [15]. Even so, developmental patterns are not identical to those of average-stature children. Differences are most noticeable in gross and fine motor domains and reflect biomechanical factors, hypotonia, and skeletal disproportion rather than intellectual impairment. For this reason, developmental assessment is best interpreted using achondroplasia-specific reference points instead of standard pediatric milestones alone [21].

Respiratory and ENT issues are among the most common extra-skeletal manifestations. Sleep-disordered breathing, including both obstructive and central sleep apnea, occurs frequently. Recurrent otitis media, middle ear dysfunction, and upper airway abnormalities are also typical [14,15,24]. Craniofacial features play an important role here. Midface hypoplasia narrows the upper airway, while mandibular changes may further contribute to airway obstruction, feeding difficulties, and temporomandibular dysfunction [19,29]. Pain and reduced physical capacity become increasingly relevant with age. Chronic pain is often reported from adolescence onward and may involve multiple anatomical regions [4,5,14]. Many adults describe fatigue, limited endurance, and difficulty walking longer distances. Gait itself is often altered, with reduced speed, increased cadence, and a flexed pattern, reflecting the combined effects of skeletal disproportion and compensatory movement strategies [16]. These factors can limit daily activities and participation.

Metabolic and cardiovascular aspects also need attention, particularly in adulthood. Achondroplasia has been associated with atypical visceral obesity, and some studies suggest an increased risk of cardiovascular disease at relatively young ages [14,16]. Other long-term concerns described in the literature include obesity, diabetes, chronic pain, and osteopenia, although these are not uniformly characterized across all studies [5]. Together, they point to the need for broader health monitoring beyond the skeletal system. Less frequently discussed are changes at the level of the intervertebral discs. FGFR3 expression has been identified in the nucleus pulposus, and structural differences such as reduced endplate size and altered annulus–nucleus relationships have been reported [7]. These findings suggest that disc development and maintenance may also be affected, potentially contributing to later spinal symptoms. Achondroplasia is therefore better understood as a multisystem condition. Respiratory, ENT, functional, metabolic, and cardiovascular features all play a role in the overall clinical picture and should be considered in long-term follow-up.

### 3.6. Developmental, Communication, and Adaptive Functioning

Developmental functioning in achondroplasia should be interpreted within the context of the condition-specific phenotype. Cognitive development is generally preserved, whereas the acquisition of gross motor milestones may be delayed because of hypotonia, relative macrocephaly, disproportionate limb length, joint laxity, reduced muscle strength, and biomechanical limitations. Sitting, standing, and independent walking may therefore occur later than in average-stature children without necessarily indicating intellectual or global developmental impairment. Developmental assessment should use achondroplasia-specific expectations whenever possible and should distinguish the anticipated motor trajectory from developmental regression, persistent asymmetry, abnormal reflexes, or marked loss of function, which may indicate cervicomedullary compression or another neurological complication.

Fine motor and self-care abilities may also be influenced by skeletal disproportion. Short upper limbs, limited elbow extension, reduced reach, brachydactyly, and difficulties with balance or mobility can affect dressing, personal hygiene, feeding, toileting, and access to objects designed for individuals of average stature. These limitations do not necessarily reflect reduced cognitive capacity, but they may delay the acquisition of practical independence. Occupational therapy, adapted equipment, environmental modifications, and age-appropriate support can facilitate autonomy at home, at school, and in the community.

Communication development may be indirectly affected by recurrent otitis media, middle-ear dysfunction, and conductive hearing loss. Reduced auditory input during early childhood can interfere with speech perception, articulation, vocabulary acquisition, classroom participation, and social interaction. Regular audiological surveillance is therefore important, particularly when speech delay, unclear articulation, reduced attention to verbal instructions, or recurrent ear infections are present. Early ENT management, hearing support, and speech and language assessment should be considered when communication difficulties are identified.

School participation may be influenced by both physical and social factors. Children may experience difficulty accessing standard furniture, toilets, laboratory equipment, transport, or activities requiring prolonged walking, climbing, or reaching. Appropriate accommodations may include adapted desks and chairs, step supports, accessible bathroom facilities, modified physical education, additional time between classrooms, and individualized educational planning. Hearing impairment, fatigue, pain, repeated medical appointments, and periods of recovery after surgery may also affect attendance and educational participation. Collaboration among families, healthcare professionals, teachers, and school support services is important for maintaining academic access and independence.

Adaptive functioning continues to evolve during adolescence and adulthood. Increasing responsibility for symptom recognition, treatment adherence, healthcare communication, mobility, personal care, education, employment, and reproductive decisions should be encouraged gradually. Environmental accessibility, assistive devices, psychological support, peer relationships, and family expectations can either facilitate or restrict autonomy. Assessment should therefore extend beyond developmental milestones and include communication, self-care, mobility, social participation, educational or occupational functioning, and the individual’s ability to navigate healthcare services. Developmental and adaptive outcomes in achondroplasia are therefore shaped by the interaction between the skeletal phenotype, neurological and sensory complications, environmental accessibility, family support, and social attitudes. A multidisciplinary approach involving pediatrics, neurology, rehabilitation, physiotherapy, occupational therapy, audiology, speech and language therapy, psychology, educational professionals, and patient organizations may help affected individuals achieve the highest possible level of autonomy and participation.

### 3.7. Age-Related Clinical Manifestations and Evolution

The clinical manifestations of achondroplasia change significantly across the lifespan. Some complications are particularly important during infancy and early childhood, especially neurological and respiratory abnormalities, whereas others become more prominent later through progressive skeletal degeneration, chronic pain, reduced mobility, and cardiometabolic risk. Understanding this chronological evolution is clinically relevant because surveillance priorities, therapeutic decisions, and functional needs vary according to age. The following table summarizes the predominant manifestations and clinical concerns typically associated with different stages of life in achondroplasia (Table 1) [9,15,21].

The life stages presented in Table 1 reflect changing clinical priorities rather than rigid chronological boundaries. During the prenatal period, the main concern is diagnostic recognition, whereas infancy represents the period of greatest neurological and respiratory vulnerability because of the risk of foramen magnum stenosis, cervicomedullary compression, hypotonia, and sleep-disordered breathing. During early childhood, developmental, sensory, and orthopedic concerns become increasingly relevant, including delayed motor milestones, thoracolumbar kyphosis, recurrent otitis media, and hearing impairment. School age introduces additional orthopedic, communication, educational, and psychosocial demands, while adolescence is increasingly characterized by chronic pain, progressive deformity, reduced exercise tolerance, and psychosocial burden. In adulthood, spinal stenosis, degenerative musculoskeletal disease, chronic pain, reduced mobility, and cardiovascular risk become more prominent. These stages overlap considerably; therefore, surveillance should remain individualized according to phenotype, previous complications, and functional status [3,9,15,16,21,24].

## 4. Complications and Comorbidities

### 4.1. Foramen Magnum Stenosis

Foramen magnum stenosis (FMS) is one of the most serious complications of achondroplasia, particularly during infancy and early childhood. Its clinical importance comes from the risk of cervicomedullary compression, which can lead to severe neurological and respiratory consequences, including sudden unexpected death if it is not recognized in time [12,20]. Some narrowing of the foramen magnum is present in almost all infants with achondroplasia and reflects impaired endochondral ossification of the skull base [12,15]. However, only a proportion of patients develop clinically significant stenosis. Current data suggest that approximately 20% of infants progress to severe forms, most often within the first two to three years of life [15]. Restricted growth of the cranial base, early closure of synchondroses, thickening of the occipital margins, and abnormal positioning of skull base elements all contribute to the reduced dimensions of the foramen magnum [12,20].

The main clinical risk is compression of the brainstem and upper cervical spinal cord, a region that contains respiratory centers and cranial nerve nuclei [20]. Infants may present with central apnea, feeding difficulties, hypotonia, or delayed motor development. Severe cases can progress to neurological deterioration or sudden death [12,15]. Reported mortality related to FMS ranges from approximately 2% to 7.5%, and mortality in young children with achondroplasia is higher than in the general population [12]. Recognition is not always straightforward. Significant anatomical compression may be present even in infants who appear clinically well [21]. Warning signs include persistent hypotonia, hyperreflexia, sustained clonus, asymmetric reflexes, delayed or regressing development, swallowing difficulties, poor weight gain, and early hand preference [12,21]. Respiratory abnormalities such as apnea, hypoxemia, or hypercapnia should also prompt urgent evaluation [15]. Screening therefore relies on more than clinical examination alone. Recommended surveillance includes repeated neurological examinations, developmental monitoring, early polysomnography, and MRI of the cranio-cervical junction, usually within the first 3–6 months of life [12,15,16]. Follow-up is most intensive in early childhood, with assessments every 3–4 months during the first year and every 3–6 months until approximately 3 years of age, after which monitoring can be individualized [12]. The decision to intervene should be based on the combined interpretation of clinical signs, imaging, sleep studies, and developmental evolution, rather than on a single abnormal result [12,15].

When clinically relevant compression is confirmed, surgical decompression is the standard treatment. The procedure usually involves suboccipital decompression, with or without cervical laminectomy and duraplasty. Indications include spinal cord compression on imaging, abnormal cerebrospinal fluid flow, central sleep apnea, neurological deficits, or progressive symptoms. Outcomes are generally favorable, with improvement reported in up to 90% of cases, although complication rates of around 20% and the possibility of reintervention remain important considerations [16]. The timing of risk is closely linked to early cranial development. In unaffected children, the foramen magnum reaches approximately 70–80% of its final size during the first year of life. In achondroplasia, growth is delayed, particularly in the transverse diameter, which helps explain why clinically significant stenosis is concentrated in infancy [20]. Experimental work suggests that modulation of FGFR3 signaling may influence cranial base development. In murine models, reducing FGFR3 expression increased foramen magnum size, raising the possibility that future disease-modifying therapies could affect this complication [5]. Whether early pharmacological treatment, including vosoritide, can meaningfully alter foramen magnum growth in clinical practice remains uncertain [24]. FMS is a potentially life-threatening but manageable complication. Its early onset and possible severity justify systematic screening, rapid recognition of warning signs, and management by teams familiar with achondroplasia.

### 4.2. Spinal Stenosis

Spinal stenosis is one of the major long-term complications of achondroplasia and becomes increasingly relevant with age, particularly from adolescence onward. Although abnormalities of the spine are present early in life, symptomatic thoracolumbar stenosis usually develops later, when congenital narrowing of the spinal canal is compounded by progressive deformity and degenerative change [20,21]. The anatomical basis is multifactorial. The spinal canal is developmentally narrow because of short, thick pedicles, reduced interpedicular distance, facet hypertrophy, thickened laminae, and a smaller anteroposterior canal diameter [16]. Over time, additional changes may further reduce the available space for the spinal cord or cauda equina. These include disc herniation, facet arthrosis, ossification of the posterior longitudinal ligament, ossification of the ligamentum flavum, thoracolumbar kyphosis, increased lumbar lordosis, anterior vertebral wedging, and ligamentous thickening [16,20].

Imaging reflects these structural constraints. Plain radiographs may show progressive narrowing of the interpedicular distance, particularly in the lumbar spine [16]. MRI provides a more complete assessment because it can demonstrate both osseous narrowing and soft tissue or degenerative contributors. Experimental models support the same anatomical explanation, showing reduced vertebral body length, interpedicular distance, and spinal canal area in achondroplasia [5]. The clinical presentation is often gradual. Patients may develop persistent low back pain, neurogenic claudication, sciatica, lower limb discomfort, paresthesias, numbness, and reduced walking distance [9,16]. Symptoms typically worsen with prolonged standing or walking and improve with rest, lumbar flexion, or squatting, a pattern characteristic of neurogenic claudication [9]. In advanced cases, bladder or bowel dysfunction, sensory loss, myelopathy, or fecal incontinence may occur [9,20]. Because symptoms may progress slowly, they can be underestimated unless they are actively sought. Some spinal manifestations, such as thoracolumbar kyphosis, appear much earlier. This deformity is common in infancy and often improves after independent walking develops, although residual deformity may persist in some children [15,21]. Later in life, thoracolumbar complications become more prominent, and symptomatic spinal stenosis is considered one of the most frequent and disabling adult complications of achondroplasia [9,20].

When symptoms suggest stenosis, investigation should not be delayed. Evaluation usually includes a neurological examination and MRI of the entire spine. If spinal cord or nerve root compression is suspected, referral to a specialist center with experience in achondroplasia is recommended [9,16]. This is clinically important because delayed diagnosis can increase the risk of irreversible neurological impairment. Severe symptomatic stenosis may require surgical treatment. Standard approaches include multilevel posterior decompression and neuroforaminal decompression, often combined with posterior fusion to reduce the risk of post-laminectomy instability. Reported outcomes are generally favorable, with symptom improvement in up to 95% of cases. Even so, the burden of surgery is significant, with some series reporting complication rates around 17% and reintervention rates around 18% [1]. In severe disease, up to one third of patients may eventually require laminectomy for symptomatic stenosis [20].

Spinal stenosis is also relevant in the context of emerging disease-modifying therapies. Expert opinion suggests that earlier molecular treatment might reduce the lifetime burden of symptomatic stenosis, although this has not yet been demonstrated clinically [22]. Experimental findings are encouraging: in murine models, reduction in FGFR3 expression improved vertebral growth, interpedicular distance, and spinal canal dimensions, with near-normalization of some vertebral parameters by adulthood [5]. These data raise the possibility that future therapies may influence axial complications, not only height. Spinal stenosis in achondroplasia is therefore produced by the interaction between congenital canal narrowing, progressive skeletal deformity, and degenerative change. Its association with pain, neurological symptoms, reduced mobility, and substantial healthcare burden makes it a key target for lifelong surveillance and timely multidisciplinary management.

### 4.3. Sleep Apnea

Sleep-disordered breathing is one of the most frequent complications of achondroplasia and may affect patients from infancy into adulthood. Both obstructive sleep apnea (OSA) and central sleep apnea can occur, sometimes in the same individual, and both contribute to morbidity and, in severe cases, increased mortality [16,21]. OSA is the predominant form. It is mainly related to craniofacial and upper airway anatomy, including midface hypoplasia, a narrow nasopharyngeal space, and adenotonsillar hypertrophy [15,21]. These features may be present early, which means that obstructive events can appear even in infancy. Reported prevalence is high, affecting approximately 50–80% of children and close to 60% of adults with achondroplasia, far above rates in the general population [21,22]. Central sleep apnea has a different mechanism and is particularly relevant in younger children. Here, the problem is not primarily airway collapse, but instability of respiratory control, often related to foramen magnum stenosis and cervicomedullary compression [12,15]. Sleep-disordered breathing in achondroplasia therefore reflects the overlap between craniofacial anatomy, skeletal narrowing, and neurological vulnerability.

Clinical presentation varies with age; older children and adults may report loud snoring, witnessed apneas, gasping during sleep, non-restorative sleep, or excessive daytime sleepiness [9]. Untreated OSA may also contribute over time to hypertension and cardiovascular strain [9,16]. In infants, signs may be less specific. Apnea, feeding difficulties, or poor weight gain may be the only clues, so clinical suspicion should remain low-threshold. Early screening is recommended because symptoms are not always reliable. Current guidance supports polysomnography within the first year of life, even in apparently asymptomatic infants [15,21]. At any age, a sleep study is indicated when symptoms suggest obstructive or central apnea. Interpretation should ideally involve clinicians familiar with achondroplasia, since abnormal findings may reflect more than one mechanism and need to be considered together with neurological examination and imaging [12].

Management depends on the dominant cause. In obstructive sleep apnea, adenotonsillectomy is often the first-line intervention and may lead to meaningful improvement in selected children [15]. Some patients require CPAP or other forms of respiratory support. When central apnea is associated with cervicomedullary compression, neurosurgical assessment is necessary, and foramen magnum decompression may be indicated. Significant respiratory complications should also be addressed before starting disease-modifying treatment such as vosoritide [24]. Sleep-disordered breathing has important perioperative implications. Patients with OSA or cervicomedullary compression are at increased anesthetic risk, which is why preoperative assessment often includes ENT evaluation and sleep investigation when indicated [16].

Attention has increasingly shifted toward earlier identification and management, not only to treat symptoms, but also to limit long-term consequences. Longitudinal evidence remains limited, but expert opinion suggests that proactive care may reduce the cumulative burden of disease [22]. In achondroplasia, sleep apnea should therefore be viewed as a common, multifactorial, and potentially serious complication requiring routine screening and coordinated multidisciplinary management.

### 4.4. ENT Complications

ENT complications are common in achondroplasia and represent an important source of morbidity, especially during childhood. Their impact may continue into adult life. Most are related to craniofacial anatomy, including midface hypoplasia, short and narrow Eustachian tubes, and relative adenotonsillar hypertrophy, which predispose to middle ear dysfunction and upper airway obstruction [21,24]. Recurrent otitis media is the most frequent ENT problem and is often associated with conductive hearing loss. It tends to appear early and can affect development in practical ways. Hearing impairment may contribute to delayed language acquisition, articulation difficulties, communication problems, and later educational challenges, which makes early surveillance important [15,21]. Chronic middle ear effusion may present with repeated infections, reduced hearing, or delayed speech development [15]. ENT morbidity is not limited to childhood. Recent data suggest that hearing problems may persist into adulthood and remain more frequent than in the general population. In one adult cohort, approximately 43% of individuals aged 16–44 years had mild hearing loss in at least one ear, supporting periodic audiological assessment even in younger adults.

Management should be guided by clinical findings. Suspected hearing loss warrants formal audiological evaluation and referral to an ENT specialist [9]. In children, tympanostomy tubes may improve conductive hearing loss and reduce recurrent otitis media, as described in clinical reports [15]. ENT-related problems also account for repeated consultations and procedures, including audiograms, adenotonsillectomy, and myringotomy, reflecting their practical burden in everyday care [14]. ENT assessment is closely linked to sleep-disordered breathing. Upper airway narrowing and adenotonsillar hypertrophy may contribute to obstructive sleep apnea or worsen existing symptoms. Patients with snoring, sleep apnea, or other upper airway concerns therefore often require ENT evaluation, including as part of preoperative assessment [16]. ENT complications in achondroplasia should be monitored actively rather than addressed only when advanced symptoms appear. Regular hearing assessment, timely ENT referral, and coordination with the multidisciplinary team are important for reducing the impact on language development, respiratory function, and quality of life.

### 4.5. Cardiovascular Risks

Cardiovascular risk in achondroplasia has received increasing attention, particularly in adult care. In adulthood, the clinical picture is influenced not only by skeletal complications, but also by metabolic factors, reduced mobility, sleep-disordered breathing, and chronic pain. Although the evidence is less extensive than for neurological or orthopedic complications, available data suggest an increased cardiovascular risk profile, especially in relation to hypertension, obesity, and reduced physical capacity [9,16].

Arterial hypertension is one of the most consistently reported concerns. It appears to be more frequent in adults with achondroplasia than in the general population, with reported prevalence rates of approximately 50% in men and 35% in women, compared with around 22% in the general population. Blood pressure monitoring should therefore be included in routine adult follow-up. Measurement technique is important: cuff size must be appropriate, and when rhizomelia or joint contractures make upper arm measurement difficult, forearm measurement may be used instead. Obesity is another relevant factor, both metabolically and functionally. Excess weight may worsen obstructive sleep apnea, hypertension, back and limb pain, spinal stenosis, and mobility limitation [9,21]. Some studies also describe atypical visceral obesity, which may further contribute to cardiovascular and systemic risk [16]. Although it is not yet clear whether obesity in achondroplasia confers the same risk of type 2 diabetes as in the general population, its ability to aggravate existing comorbidities is clinically important [9].

Nutritional monitoring, a balanced diet, and regular physical activity are therefore recommended, even though disease-specific criteria for obesity classification and management remain limited. Exercise advice should be realistic and individualized, as pain, limb deformity, spinal stenosis, reduced endurance, and neurological risk may all restrict participation. Chronic pain contributes indirectly to cardiovascular risk by reducing activity levels. Around 65–70% of adults with achondroplasia report chronic pain, which may affect self-care, work, social participation, and exercise tolerance [9]. This can create a self-reinforcing cycle: pain limits movement, reduced movement promotes deconditioning and weight gain, and both may further increase cardiometabolic risk.

The clinical relevance of this issue is supported by observations that cardiovascular mortality may occur earlier in achondroplasia than in the general population, in some reports beginning as early as the third decade of life [16]. Cardiovascular surveillance should therefore be incorporated into long-term care from early adulthood rather than postponed until later life. Newer therapies also require attention to cardiovascular parameters. Guidance on vosoritide notes that patients with underlying cardiovascular disease or predisposition to hypotension may be at greater risk of symptomatic blood pressure reduction. Blood pressure and heart rate monitoring are therefore included among routine safety assessments during treatment [24]. Cardiovascular risk in achondroplasia is best understood as the result of interacting factors. Hypertension, obesity, sleep-disordered breathing, chronic pain, and reduced mobility may reinforce one another, supporting a preventive approach based on regular monitoring, early intervention, and individualized lifestyle guidance.

### 4.6. Oral and Dental Complications

Achondroplasia has important craniofacial and dentofacial manifestations that may affect oral function, occlusion, airway anatomy, and dental management. Although the condition is primarily considered a skeletal dysplasia, several studies emphasize its relevance in dental and orthodontic practice because abnormalities of the cranial base and midface may substantially influence oral development [3,8,17]. The craniofacial phenotype commonly includes relative macrocephaly, frontal bossing, a depressed nasal bridge, and maxillary or midfacial hypoplasia [3,8]. A short posterior cranial base and otolaryngeal dysfunction have also been described [8]. These abnormalities are not purely cosmetic; they may contribute to upper airway obstruction, recurrent sinusitis, otitis media, sleep-disordered breathing, and dental malocclusion [3,8]. Midfacial hypoplasia appears to play a central role in the development of many oral manifestations, particularly because mandibular growth is often relatively preserved. Chhabra et al. (2016) [8] suggested that this imbalance reflects impaired endochondral ossification affecting the maxilla and skull base, whereas mandibular growth is less severely affected because condylar cartilage derives largely from periosteal chondrogenesis.

The most frequently reported intraoral findings include macroglossia, tongue-thrust swallowing, Class III malocclusion, posterior crossbite, anterior reverse overjet, anterior open bite, crowding, and retained primary teeth [3,8,17]. Constricted maxillary arches and skeletal Class III relationships are particularly characteristic and are generally explained by maxillary hypoplasia associated with a relatively normal mandible [8,17]. Several authors also described concave facial profiles, saddle nose deformity, and severe midface deficiency in affected patients [3,8]. Functional consequences may include speech difficulties, mouth breathing, altered swallowing patterns, and impaired mastication, especially when severe malocclusion and macroglossia coexist [8]. Dental development appears to be more variable than craniofacial morphology. Some reports describe delayed eruption of permanent teeth, whereas others found eruption patterns compatible with chronological age [3,17]. In the cases reported by Nanmaran et al. (2023) [17] and Chhabra et al. (2016) [8], panoramic imaging demonstrated normal tooth number, morphology, and crown development, including third molars. These findings suggest that the primary abnormalities in achondroplasia are mainly craniofacial and orthodontic rather than intrinsic defects of tooth structure.

The relationship between achondroplasia and dental caries should be interpreted cautiously. Current evidence does not support the conclusion that achondroplasia directly increases caries susceptibility. However, several factors associated with the condition, including malocclusion, mouth breathing, macroglossia, impaired oral hygiene, and dietary habits, may contribute to increased individual caries risk in some patients [3,17]. Gingivitis related to plaque accumulation and poor oral hygiene has also been reported [3]. Orthodontic management may be particularly challenging in achondroplasia. Growth modification strategies cannot always be applied in the same way as in unaffected children because skeletal growth potential is altered [8]. Early orthodontic assessment is therefore recommended, especially for Class III malocclusion and maxillary constriction. The American Academy of Pediatrics has recommended orthodontic review after 5 years of age in children with achondroplasia [8].

Dental treatment itself may require important precautions. Short stature, spinal deformity, macrocephaly, limited neck extension, craniocervical instability, foramen magnum stenosis, and airway abnormalities can complicate positioning and airway management during dental procedures [3,8]. These issues become especially relevant in uncooperative children or when sedation and general anesthesia are required. Difficult airway anatomy, including a relatively narrow nasopharynx and larynx, anteriorly positioned epiglottis, small thorax, and potential cervical instability, may increase anesthetic risk [3,8]. For this reason, careful preoperative evaluation, attention to head positioning, and appropriate airway planning are recommended before sedation or anesthesia. Achondroplasia should therefore be recognized not only as a skeletal disorder, but also as a condition with clinically important oral and craniofacial implications requiring multidisciplinary dental and orthodontic care (Table 2).

The complications summarized in Table 2 differ substantially in timing, clinical urgency, and strength of evidence. Foramen magnum stenosis and cervicomedullary compression require particular attention during infancy, whereas symptomatic spinal stenosis becomes increasingly relevant during adolescence and adulthood. Sleep-disordered breathing and ENT complications may occur across several developmental stages and therefore require longitudinal surveillance. Cardiovascular and metabolic risk becomes particularly relevant in adult care, while dental and craniofacial abnormalities may influence feeding, airway function, communication, and orthodontic management from childhood onward. The screening and management approaches presented in the table integrate observational evidence with published clinical recommendations; where direct empirical evidence remains limited, recommendations are predominantly consensus-based [3,8,9,12,15,16,17,20,21,24].

## 5. Diagnosis

### 5.1. Clinical Diagnosis

In many cases, achondroplasia can be recognized clinically by bringing together the typical phenotype, the clinical context, and when available supportive imaging. After birth, the diagnosis is often suspected quite early, sometimes within the first days of life, simply because the combination of features is so characteristic [15,21]. Certain signs tend to stand out. Macrocephaly, frontal bossing, midface hypoplasia, and disproportionate shortening of the limbs especially with a rhizomelic pattern are among the most suggestive findings. The trident hand configuration is another well-known clue [15]. As the child grows, the picture becomes more complete: short stature becomes more evident, limb alignment may change, and other skeletal features gradually reinforce the diagnosis [29]. In straightforward cases, this constellation is often enough to orient the clinician quickly.

Still, things are not always that clear at the beginning. Some newborns have only subtle features, and length at birth may fall within ranges that do not immediately raise concern. Midface hypoplasia or limb disproportion may only become apparent on closer examination or over time, as growth patterns diverge from what is expected [15]. This early variability explains why, in some cases, diagnosis is confirmed later through imaging or genetic testing rather than at first inspection. Prenatal recognition adds another layer of difficulty. Ultrasound findings can be quite subtle, especially before 24 weeks of gestation [21]. It is not unusual for the second-trimester anomaly scan to appear normal. Suspicion tends to arise later, often in the third trimester, when features such as femoral shortening or relative macrocephaly become more evident [15,21]. In selected situations, single-gene non-invasive prenatal testing (NIPT) can support earlier detection, although its use depends on context and availability [15]. Clinical evaluation is not limited to identifying the phenotype. In infancy, it plays a key role in detecting early complications, particularly those related to foramen magnum stenosis. This means going beyond a general examination: neurological history, muscle tone, presence of clonus, asymmetry, head circumference trends, and developmental progress all need attention [12]. Sometimes, small findings taken together are what raise concern. Overall, the clinical diagnosis of achondroplasia relies on a recognizable pattern, but recognizing that pattern is not always immediate. Experience helps, but so does careful observation over time. In typical cases, the phenotype points clearly toward the diagnosis; in less obvious ones, imaging and genetic testing help complete the picture.

### 5.2. Imaging

Imaging is central to the evaluation of achondroplasia. It supports the clinical diagnosis, documents the skeletal phenotype, and helps identify neurological or orthopedic complications that may not be apparent on examination alone [15,21]. Conventional radiography is especially useful in the neonatal period and early childhood. After birth, a babygram or targeted skeletal radiographs can rapidly show the typical features of the disorder [15]. The most characteristic findings include short, square iliac bones, narrow sacrosciatic notches, and progressive narrowing of the lumbar interpedicular distance from L1 to L5, one of the classic radiographic signs of achondroplasia [21]. Vertebral abnormalities, short femoral necks, and characteristic changes in the long bones may also support the diagnosis [29]. MRI has a different role, but is equally important. It is the preferred method for assessing the foramen magnum and cranio-cervical junction, particularly when cervicomedullary compression is a concern [12,21]. This matters most in infancy, when clinical examination alone may miss severe narrowing. Published data suggest that relevant cases of compression can be overlooked if evaluation relies only on symptoms or cardiorespiratory studies, which explains why MRI is included in screening strategies [12].

Current recommendations support routine MRI of the cranio-cervical region at around 3–6 months of age, with repeat imaging guided by symptoms or abnormalities found on other assessments [12]. In infants and young children, this usually includes brain MRI and imaging of the cervicomedullary junction; in selected cases, full cervical MRI with flexion and extension views may be considered [15]. Interpretation should ideally be performed by clinicians and radiologists familiar with achondroplasia, since both underdiagnosis and over-interpretation are possible without specific experience [12]. The Achondroplasia Foramen Magnum Score (AFMS) is one useful MRI-based tool. It grades stenosis according to its effect on neural structures rather than simple anatomical size alone. AFMS 0 indicates a normal cranio-cervical junction; AFMS 1, narrowing with cerebrospinal fluid still visible around the cord; AFMS 2, loss of cerebrospinal fluid space without cord deformation; AFMS 3, cord deformation; and AFMS 4, T2 signal change at the cranio-cervical junction [20]. This kind of structured assessment can help with follow-up and neurosurgical decision-making.

For spinal disease beyond the foramen magnum, plain radiographs remain useful for assessing interpedicular distance and vertebral alignment. MRI is preferred when symptoms suggest spinal stenosis, such as low back pain, neurogenic claudication, or signs of cord or nerve root compression. Imaging is also important in monitoring orthopedic deformities, including persistent thoracolumbar kyphosis, scoliosis, axis deviation, and in planning corrective or lengthening procedures [16]. There are practical issues as well, especially in infants. MRI may require general anesthesia, but in young infants the feed-and-wrap technique can sometimes be used, usually up to around 6 months of age, avoiding some of the risks associated with sedation [12]. In practice, radiography and MRI answer different but complementary questions. Radiographs define the skeletal pattern, while MRI is essential for complications involving the cranio-cervical junction and spine, particularly when neurological risk is suspected.

### 5.3. Genetic Testing

Genetic testing is an important part of diagnosing achondroplasia, especially when the phenotype is incomplete, atypical, or first suspected prenatally. In many postnatal cases, the clinical picture is already highly suggestive, but molecular testing provides confirmation and helps distinguish achondroplasia from other skeletal dysplasias or related FGFR3 disorders with overlapping features [7,21]. In postnatal practice, testing usually focuses first on the recurrent FGFR3 variants c.1138G>A and c.1138G>C, both of which lead to the p.Gly380Arg substitution seen in the great majority of cases [15]. Targeted methods, including PCR-based testing for these common variants, are therefore a practical first-line option. If targeted testing is negative but clinical suspicion remains strong, full FGFR3 sequencing is recommended to identify rarer pathogenic variants and clarify the differential diagnosis [29]. Genetic confirmation has become particularly important with the introduction of disease-modifying therapy. Before vosoritide is started, the diagnosis should be confirmed molecularly. If a clinical diagnosis was made previously but the genetic report is unavailable, repeat targeted testing for achondroplasia-associated FGFR3 variants is recommended. A negative result should prompt further diagnostic evaluation rather than treatment initiation, with referral to medical genetics when needed. For newly diagnosed patients, genetic consultation also provides counseling, education, and appropriate classification within the broader group of FGFR3-opathies [24].

Prenatal testing has also become more relevant. Traditional invasive methods, such as chorionic villus sampling and amniocentesis, allow direct analysis of FGFR3 when ultrasound findings or family history raise suspicion [29]. More recently, expanded single-gene non-invasive prenatal testing has made detection of c.1138G>A and c.1138G>C possible in selected pregnancies. This approach has an important limitation: it is reliable only when the pregnant parent is not affected, because otherwise the test cannot confidently distinguish a maternal variant from a fetal one [15]. Genetic testing is especially useful when the presentation is unusual. Reviews of the FGFR3 mutational spectrum show that molecular analysis helps not only to confirm classic achondroplasia, but also to separate it from other phenotypes caused by different variants in the same gene [7]. In this way, testing does more than name the diagnosis; it places the patient correctly within the wider spectrum of FGFR3-related disorders. Genetic testing is therefore now embedded in the diagnostic pathway. It confirms typical cases, helps resolve atypical ones, supports prenatal diagnosis, and is increasingly necessary for treatment decisions (Table 3).

The diagnostic approaches summarized in Table 3 are complementary rather than interchangeable. Prenatal ultrasonography primarily raises diagnostic suspicion, whereas postnatal clinical and radiographic findings define the characteristic skeletal phenotype. MRI is primarily used to identify clinically relevant cranio-cervical and spinal complications. Targeted FGFR3 testing provides molecular confirmation in most typical cases, while extended genetic testing is particularly relevant when targeted analysis is negative or the phenotype is atypical. The increasing availability of disease-modifying therapy has further strengthened the clinical importance of molecular confirmation [7,12,15,21,24,29].

## 6. Management

### 6.1. Multidisciplinary Care

Achondroplasia is a multisystem condition with care needs that begin before birth and continue throughout adult life. Because its complications involve several anatomical and functional systems, management cannot be reduced to a single specialty. Current evidence supports a coordinated model of care that aims not only to detect complications, but also to preserve function, independence, and quality of life over time [13,14,21].

In childhood, the preferred setting is a specialized center for achondroplasia or skeletal dysplasias, where assessment and follow-up can be organized in a structured way. Depending on age and clinical needs, the team may include a pediatrician, clinical geneticist, pediatric endocrinologist, neurologist or neurosurgeon, ENT specialist, pulmonologist or sleep specialist, orthopedic surgeon, anesthesiologist, physiotherapist, rehabilitation specialist, specialized nurse, psychologist, and nutrition specialist [16]. The purpose of such a team is not only to respond to established complications, but also to anticipate predictable risks, including foramen magnum stenosis, sleep-disordered breathing, ENT disease, limb deformities, chronic pain, and functional limitation [13,21]. A coordinating clinician is essential in this model. Ideally, each patient should have one professional who organizes investigations, follow-up, and referrals, especially when care is not provided within a tertiary center [15]. Since the introduction of vosoritide, the pediatric endocrinologist may often assume this role. Even so, growth-promoting therapy represents only one part of care and should not replace broader multidisciplinary follow-up [15,24].

Coordination may begin as early as the prenatal period. When achondroplasia is suspected antenatally, early referral to a skeletal dysplasia specialist, pediatrician, geneticist, fetal medicine unit, and, when appropriate, pediatric endocrinologist is recommended to plan delivery and postnatal care [15]. This is particularly relevant because the first months of life are a high-risk period, when neurological and respiratory complications must be assessed through combined clinical, imaging, and functional evaluation [12,24]. Pharmacological treatment also needs to be integrated into this wider care pathway. Before starting vosoritide, patients should be assessed for eligibility, disease severity, and comorbidities that may affect safety or response. In infants, sleep assessment and MRI of the cranio-cervical junction are recommended before treatment; significant cranio-cervical stenosis, spinal deformity, or severe sleep-disordered breathing should be managed first. Monitoring during therapy should include more than height and growth velocity, since clinical, radiological, functional, laboratory, and cardiovascular assessments may all be relevant, particularly in early childhood [24].

Modern management also includes functional and psychosocial care. The orthopedic literature increasingly emphasizes that treatment goals should not be limited to height gain or correction of deformity. Daily function, participation, prevention of long-term complications, and outcomes valued by patients and families are equally important [16]. This perspective is clinically important because pain, reduced mobility, fatigue, difficulties with self-care, and social challenges may persist even when major medical complications are controlled [14,21]. The family should be considered part of the care context. Parents often manage frequent appointments, complex treatment decisions, and logistical pressures, all of which may add emotional strain. Periodic psychosocial screening of caregivers, access to psychological support, referral to support groups, and anticipatory guidance can therefore be useful components of management [1].

Adult care remains one of the weaker points in many healthcare systems. Pediatric follow-up is often structured, but many patients are lost after transition, and adult management may rely mainly on primary care. A pragmatic model has been proposed in which a primary care clinician coordinates follow-up and refers patients to specialists familiar with achondroplasia when warning symptoms appear. Although this is less comprehensive than a dedicated multidisciplinary center, it may be more feasible across different healthcare settings and can reduce delays in managing complications [9]. Transition should be treated as a clinical process, not simply as the end of pediatric follow-up. Patients need gradual transfer of responsibility, education about possible complications and red flags, and clear information on how to access specialist care [9,16]. Without this preparation, complications such as spinal stenosis or obstructive sleep apnea may be recognized late, with avoidable functional decline.

**Figure 1:**
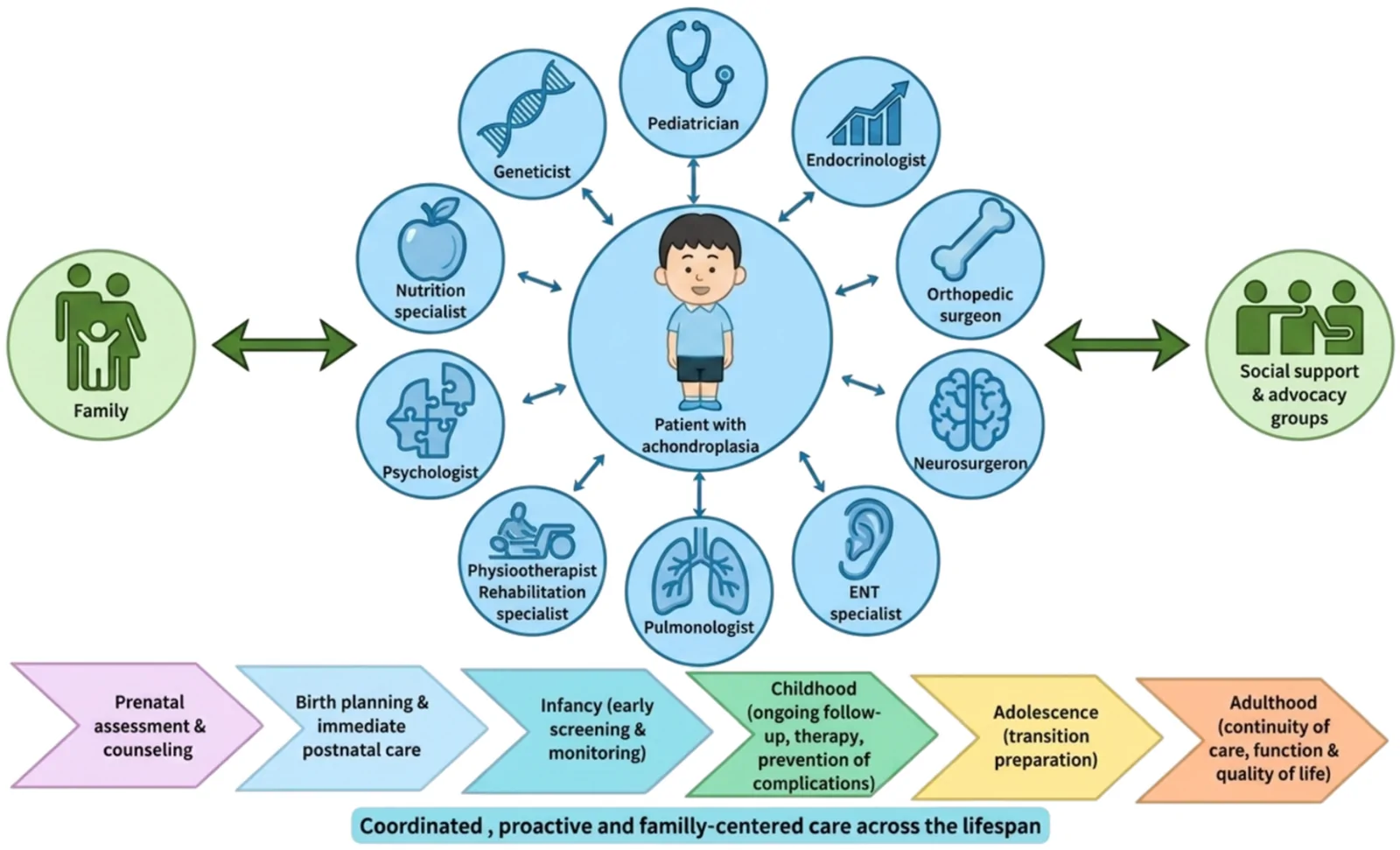
Multidisciplinary care model in achondroplasia across the lifespan. Management requires coordinated involvement of multiple specialties, early surveillance for complications, family-centered support, and continuity of care from infancy to adulthood. Created in BioRender. Văruț, R.M. (2026); https://app.biorender.com/illustrations/canvas-beta/6a5e441930e214810ad20b2b and adapted from references [1,9,15,16,21,24].

Multidisciplinary input is also essential when orthopedic or spinal surgery is considered. These procedures should be performed in experienced centers, after interdisciplinary preoperative assessment and with coordinated postoperative follow-up [2,16,21]. This is even more relevant now that disease-modifying therapies may influence growth patterns and potentially alter the timing of guided growth, axis correction, or limb lengthening [6,16]. A flexible but well-coordinated multidisciplinary model is central to achondroplasia care. Its aim is not only to treat complications, but also to preserve function, reduce the burden on patients and families, and maintain continuity of care across the lifespan (Figure 1).

Figure 1 illustrates the proposed lifespan model of multidisciplinary care. The patient is positioned at the center of the care network, reflecting coordinated input from medical, surgical, rehabilitation, nutritional, psychological, and genetic specialties. Family members and social or advocacy networks represent additional components of care because they influence treatment implementation, autonomy, adaptation, and psychosocial well-being. The lower pathway illustrates the progressive shift from prenatal counseling and birth planning to intensive surveillance during infancy, continuing multidisciplinary management during childhood, transition preparation during adolescence, and continuity of specialist care in adulthood.

### 6.2. Anthropometric Measurements and Growth Monitoring

Anthropometric monitoring is an essential component of the clinical follow-up of patients with achondroplasia. Because the condition is characterized by disproportionate short stature and altered body proportions, standard population growth charts are not adequate for accurate assessment. Several authors emphasize that syndrome-specific growth references are necessary for both clinical practice and research, particularly during infancy and childhood, when growth patterns differ substantially from those of average-stature individuals [10,18]. Monitoring growth in achondroplasia is challenging because the disorder affects not only overall stature, but also the relative proportions of the body. Although birth length may be close to the normal range, growth velocity progressively diverges from that of the general population during early childhood. Neumeyer et al. (2021) [30] reported that height commonly decreases to approximately −5 standard deviation scores during childhood and may reach around −6 SDS in adulthood when compared with standard population references. The disproportion primarily results from marked shortening of the limbs, while trunk length is relatively preserved. For this reason, growth assessment should not rely solely on height measurements. Current recommendations support longitudinal evaluation of several anthropometric variables, including length or standing height, weight, head circumference, sitting height, arm span, limb segment lengths, and body proportion ratios [11,18]. Repeated measurements are important not only for monitoring normal growth patterns, but also for detecting complications, evaluating nutritional status, assessing response to pharmacological therapies or limb-lengthening procedures, and identifying atypical clinical evolution.

Large achondroplasia-specific growth datasets have recently improved the availability of standardized references. The CLARITY study developed updated growth curves for stature/length-for-age, weight-for-age, head circumference-for-age, and weight-for-height using more than 37,000 anthropometric measurements obtained from over 1300 individuals with achondroplasia in the United States [10]. The authors concluded that diagnosis-specific growth curves are essential for routine clinical care because interpretation based on general population references may be misleading. Head circumference monitoring is particularly important during infancy and early childhood. Children with achondroplasia commonly have relative macrocephaly and accelerated cranial growth during the first years of life. Hoover-Fong et al. (2021) [31] observed that approximately 90% of median head circumference at five years of age is already reached by around 12–13 months. Similarly, Neumeyer et al. (2021) [30] emphasized that rapid increases in head circumference may indicate disturbances in cerebrospinal fluid circulation or evolving hydrocephalus and should prompt further investigation. Current recommendations therefore support regular measurement of head circumference at least until five to six years of age [11].

Assessment of body proportions is also important because disproportion is a defining feature of the condition. Neumeyer et al. (2021) [30] showed that arm span and leg length are substantially reduced compared with the general population from early childhood onward, while sitting height is relatively less affected. As a result, the sitting height-to-height ratio becomes markedly increased. Measurements such as sitting height, crown-rump length, arm span, and lower limb length may therefore provide clinically useful information regarding skeletal disproportion and treatment response [11,18]. Accurate anthropometric evaluation requires standardized and reproducible measurement techniques. Hoover-Fong et al. (2025) [31] noted that conventional methods may be more difficult in achondroplasia because of skeletal deformities, genu varum, tibial bowing, spinal abnormalities, joint contractures, or hypotonia. To improve reproducibility, the authors recommend the use of standardized local protocols, calibrated equipment, and trained personnel. Whenever possible, measurements should be performed consistently by the same examiner or by a limited number of trained healthcare professionals. Specific attention is also required during the transition from recumbent length to standing height measurements. For children younger than two years, length should be measured in the supine position, whereas standing height becomes preferable once the child can stand independently. Hoover-Fong et al. (2025) [31] recommend obtaining both measurements between two and three years of age because a difference of approximately one centimeter may occur between recumbent and standing measurements.

Body mass assessment in achondroplasia also requires caution. Because BMI is strongly influenced by height and body proportions, its interpretation using general population references may overestimate obesity risk. Both Neumeyer et al. (2021) [30] and Hoover-Fong et al. (2025) [31] emphasized that BMI may be artificially elevated due to short limb length and disproportionate body composition. For this reason, weight-for-height references and diagnosis-specific BMI curves may provide more clinically meaningful information than general BMI cut-offs. Waist circumference and waist-to-hip ratio may additionally contribute to metabolic risk assessment, particularly in adolescents and adults [11]. Anthropometric monitoring remains relevant even after longitudinal growth is complete. In adults with achondroplasia, repeated measurements may help identify progressive spinal deformities, loss of stature, obesity-related complications, or changes associated with degenerative disease. Recommended adult measurements include at minimum weight and standing height, with additional measures such as waist circumference, hip circumference, neck circumference, arm span, and upper limb measurements used when clinically indicated [11]. Overall, anthropometric assessment in achondroplasia should be viewed as a dynamic and multidimensional process rather than a simple measurement of height. Syndrome-specific growth charts, systematic evaluation of body proportions, careful interpretation of weight-related indices, and standardized measurement techniques are all necessary for appropriate long-term surveillance and individualized patient care [10,11,18].

### 6.3. Pediatric Management

Pediatric management of achondroplasia should begin early, as the first years of life carry the highest risk for severe neurological and respiratory complications. Children should ideally be followed by a multidisciplinary team coordinated by a clinician experienced in achondroplasia, particularly during the first two years, when surveillance must be frequent and focused on complications that may become life-threatening [12,21].

During infancy, follow-up is usually recommended every 2–4 months in the first year, then every 3–6 months according to clinical status and individual risk. The main priorities are detection of foramen magnum stenosis, hydrocephalus, and sleep-disordered breathing, alongside monitoring of growth and neurodevelopment [15,21]. European Achondroplasia Forum recommendations support frequent clinical assessment until 3 years of age, including routine MRI of the cranio-cervical junction at 3–6 months, with repeat imaging when clinical or functional findings raise concern for cervicomedullary compression [12].

The focus of pediatric follow-up changes with age. In infancy, surveillance is directed mainly toward foramen magnum stenosis, cervicomedullary compression, central or obstructive apnea, hydrocephalus, feeding difficulties, and motor development. During early childhood, recurrent otitis media, hearing impairment, thoracolumbar kyphosis, delayed motor milestones, growth monitoring, and family education become particularly relevant. In later childhood and adolescence, management increasingly focuses on obstructive sleep apnea, progressive lower-limb deformities such as genu varum, pain, weight gain, reduced physical activity, functional independence, and psychosocial adaptation [12,16,21]. Growth assessment requires achondroplasia-specific charts. Length or height, weight, and head circumference should be interpreted using condition-specific references, since standard charts for average-stature children may be misleading. Conventional BMI charts are also poorly suited to this population, and dedicated tools are preferred for assessing body proportions and nutritional status [15,21]. This distinction helps separate expected disease-related growth patterns from changes that may indicate complications or additional pathology.

Developmental assessment needs the same disease-specific approach. Children with achondroplasia often acquire gross motor milestones later than average-stature peers, but this delay is usually part of the expected developmental pattern rather than evidence of neurological disease. Assessment should therefore be based on achondroplasia-specific expectations [13,15]. Delayed independent walking may fall within the typical range, whereas regression, absence of progress, or marked deviation from expected patterns should prompt neurological and imaging evaluation [24]. Physiotherapy should be individualized rather than applied routinely to every child. If an infant reaches expected milestones for achondroplasia and shows no signs of neurological or orthopedic deterioration, routine physiotherapy may not be necessary. It becomes more relevant in the presence of thoracolumbar kyphosis, significant motor delay, or during perioperative and postoperative care, particularly after orthopedic procedures such as limb lengthening [15,16].

Parental education is another central element of pediatric care. Families need practical guidance on handling and positioning, since some routine practices may worsen spinal deformity. Early unsupported sitting and positions that accentuate thoracolumbar kyphosis should be avoided to reduce the risk of progression from a flexible to a fixed deformity [15,21]. Safe transport should also be discussed, including the risks of prolonged use of standard car seats and the possible need for a car bed when head control is insufficient [15]. Screening for major complications remains a core part of early care. Polysomnography, repeated neurological examinations, and cranio-cervical MRI are used to identify foramen magnum stenosis, central or obstructive sleep apnea, and other causes of cervicomedullary compression [12,15,21]. When symptomatic compression is confirmed, foramen magnum decompression is indicated, with the decision made by an experienced multidisciplinary team [12,21].

After infancy, surveillance gradually shifts toward complications that become more evident with growth, including obstructive sleep apnea, chronic otitis media, progressive lower limb deformities, and possible spinal stenosis [13,15]. Orthopedic monitoring becomes more important, and recommendations for rehabilitation and physical activity should be adapted to limb alignment, joint laxity, functional capacity, and neurological risk. Weight monitoring should not be overlooked. Body proportions make standard assessment difficult, but excessive weight gain can worsen sleep apnea, orthopedic problems, and functional limitation. Physical activity should be encouraged, although advice must be realistic and tailored to each child’s deformities, neurological status, endurance, and overall level of function [16]. The introduction of disease-modifying treatment has added complexity to pediatric management. Vosoritide should be integrated into a broader care plan rather than used as a stand-alone intervention. Before treatment, children should be assessed for eligibility, severe complications, and factors that may affect growth response or safety. In infants, sleep studies and cranio-cervical MRI are recommended before initiation, and major issues such as cervicomedullary compression, significant spinal deformity, or severe sleep-disordered breathing should be managed first. After starting therapy, follow-up usually includes an early review at 4 weeks, then assessments at 6 months and annually; children under 24 months require closer monitoring, preferably in specialized centers [24]. Long-term planning is particularly important when pharmacological therapy is considered alongside orthopedic procedures. Recent consensus statements emphasize the need to plan according to age and pubertal stage, define short- and long-term goals, assess patient and family motivation, and discuss risks and benefits realistically [6]. These decisions may influence growth, function, and quality of life well beyond childhood.

Pediatric care should also include the family. Caregivers often manage frequent visits, repeated investigations, complex decisions, and logistical strain. In some pediatric cohorts, nearly all children experienced significant medical events, required multiple specialist consultations, and a large proportion underwent at least one surgical procedure [14]. Parental quality of life may also influence how the child’s quality of life is perceived, supporting a family-centered model that includes psychosocial support as part of routine care [1]. Pediatric management is therefore a continuous and adaptive process. It combines surveillance, prevention of complications, functional support, family education, and careful integration of new therapies within a coordinated care framework.

### 6.4. Adult Management

Adult management should be viewed as a continuation of pediatric care, rather than as a separate or less intensive phase. Complications do not stop after childhood; in many patients, they become more clinically relevant with age. Physical function and general health may decline progressively, particularly from the third or fourth decade onward, which makes regular follow-up and early recognition of disease-specific complications essential [13,21]. One of the main challenges is the loss of continuity after transition from pediatric services. Childhood care is often structured and multidisciplinary, whereas adult care may become fragmented, with many patients relying mainly on primary care. The recent literature therefore emphasizes the value of planned transition and of a coordinating clinician who can identify warning symptoms and refer the patient to appropriate specialists when needed [9,16].

Clinically stable adults may not require frequent specialist visits, but they should be well informed about symptoms that require reassessment. The European Achondroplasia Forum has proposed a patient-held checklist to support self-monitoring and routine consultations. This tool is intended to improve autonomy, guide discussions with primary care clinicians, and accelerate referral when symptoms suggest complications such as spinal stenosis or obstructive sleep apnea [9]. Neurological and degenerative musculoskeletal complications deserve particular attention. Symptoms suggestive of spinal stenosis should be recognized early and investigated promptly, given their potential impact on pain, mobility, and long-term function [16,21]. Although a new diagnosis of foramen magnum stenosis in adulthood is uncommon, previously undetected cases should still be assessed individually by a multidisciplinary team familiar with achondroplasia, since evidence in this area remains limited [12]. Pain is one of the central issues in adult care. It is common, often multifactorial, and may arise from skeletal deformity, spinal stenosis, joint overload, altered gait mechanics, or degenerative disease. Management should not rely only on analgesics. A broader assessment is usually needed, integrating orthopedic, neurological, functional, rehabilitation, and, when appropriate, psychological input [16,21]. This is clinically relevant because chronic pain can affect independence, work capacity, social participation, and mental health.

Routine adult follow-up should also include cardiovascular and metabolic assessment. Blood pressure, body weight, physical activity, hearing, pain, assistive needs, psychosocial health, and genetic counseling are all included in the EAF approach to adult monitoring [9]. A healthy diet and regular physical activity are recommended not only for weight control, but also for maintaining function and general health [21]. Physical activity may have a particularly useful role. Available data suggest that moderate activity is associated with better general health, vitality, and physical functioning. Promotion of adapted activity may be especially relevant for inactive individuals and possibly for women, who appear to be at higher risk of poorer psychosocial and functional outcomes [4]. Recommendations still need to be individualized, taking into account pain, neurological status, deformities, and functional limitations. Psychosocial support should be part of routine adult care. Adults with achondroplasia may face stigma, accessibility barriers, functional restrictions, and the psychological burden of living with a visible chronic condition. Identifying the need for environmental adaptations or assistive devices can improve autonomy, and occupational therapy referral may be useful when practical limitations are present [9,21].

Genetic counseling remains relevant in adulthood, especially in the reproductive context. Routine consultations should include discussion of inheritance, reproductive options, and pregnancy when appropriate, with referral to a clinical geneticist or genetic counselor if needed [9]. This supports informed decision-making and addresses questions that may not have been relevant earlier in life. Severe orthopedic complications require specialist input. Advanced osteoarthritis, disabling pain, or neurological deterioration related to spinal stenosis should be managed in centers experienced in achondroplasia. Major procedures such as spinal surgery or hip and knee arthroplasty require careful planning because of anatomical differences, anesthetic risks, and greater technical complexity [16]. Adult care therefore depends not only on periodic assessment, but also on a reliable referral pathway to specialized services. Adult management should remain proactive and individualized. Even if follow-up is more flexible than in childhood, it should not become passive. Adults with achondroplasia need a clear medical pathway, awareness of red flags, regular review of function and psychosocial health, and access to specialist centers when clinical complexity requires it.

### 6.5. Transition from Pediatric to Adult Healthcare

Evidence specifically evaluating structured transition programs in achondroplasia remains limited. Consequently, several recommendations in this section are derived from achondroplasia consensus guidance, expert recommendations for adult follow-up, and the broader transition literature for rare or chronic conditions rather than from comparative trials of achondroplasia-specific transition models.

Transition from pediatric to adult healthcare should be planned as a gradual process rather than treated as a single administrative transfer. Preparation should begin during early adolescence and should progressively involve the young person in understanding the diagnosis, recognizing warning symptoms, communicating with healthcare professionals, managing appointments, and participating in treatment decisions. The pace of increasing responsibility should be individualized according to developmental maturity, functional ability, family context, and healthcare needs. Before transfer, the pediatric team should prepare a structured medical summary that includes the molecular diagnosis, previous cranio-cervical and spinal imaging, neurological and respiratory history, sleep-study results, hearing assessments, orthopedic deformities and procedures, growth and treatment history, pain and mobility status, cardiovascular risk factors, allergies, assistive devices, and current psychosocial or educational needs. The summary should also identify unresolved problems and specify the recommended adult surveillance plan.

A named clinician should coordinate the transition and confirm that appropriate adult services are available. Depending on local resources, this role may be assumed by a primary care physician, internist, clinical geneticist, endocrinologist, rehabilitation specialist, or clinician working within a rare-disease center. Clear referral pathways should be established for neurology, neurosurgery, orthopedics, respiratory and sleep medicine, ENT and audiology, cardiology, pain management, rehabilitation, psychology, and genetic counseling. Transition planning should also address education, employment, mobility, accessibility, reproductive counseling, independent living, and health insurance or reimbursement when relevant. The young adult should receive practical information about symptoms requiring urgent assessment, including progressive weakness, sensory changes, reduced walking tolerance, bladder or bowel dysfunction, worsening sleep-disordered breathing, severe pain, and loss of functional independence. Successful transition should be evaluated through continuity of follow-up, patient knowledge, self-management skills, access to specialists, and patient-reported experience rather than by transfer completion alone [21,30,31,32,33] (Table 4).

Table 4 should be interpreted as a practical transition framework rather than as a formally validated achondroplasia-specific transition protocol. Achondroplasia-specific evidence regarding structured transition interventions remains limited. The proposed domains therefore integrate achondroplasia consensus guidance and adult follow-up recommendations with broader evidence concerning transition in adolescents and young adults with rare or chronic conditions. Particular attention is given to information that may be lost during transfer, including previous cranio-cervical and spinal imaging, sleep-disordered breathing, orthopedic procedures, pain and mobility status, hearing impairment, psychosocial needs, and unresolved clinical problems. Continuity of care, rather than administrative transfer alone, should be considered a key indicator of successful transition [21,32,33,34,35].

## 7. Disease-Modifying Therapy in Clinical Practice

### 7.1. Vosoritide: Eligibility and Clinical Expectations

Vosoritide has introduced the first disease-modifying pharmacological treatment for children with achondroplasia and represents an important transition from exclusively supportive management toward targeted modification of impaired skeletal growth. Its clinical use is intended for children with genetically confirmed achondroplasia who have open growth plates and residual growth potential. Treatment eligibility should be assessed individually, considering chronological and skeletal age, growth pattern, comorbidities, neurological and respiratory status, cardiovascular risk, and the ability of the patient and family to comply with daily administration and long-term monitoring.

Before treatment initiation, the diagnosis should be confirmed molecularly, and baseline evaluation should include anthropometric measurements, growth velocity, body proportions, blood pressure, cardiovascular history, neurological examination, and assessment of major complications. In infants and young children, particular attention should be paid to foramen magnum stenosis, cervicomedullary compression, spinal deformity, and sleep-disordered breathing. Significant neurological or respiratory complications should be evaluated and managed before treatment is initiated [15,24].

The principal demonstrated benefit of vosoritide is an increase in annualized growth velocity. However, expectations should remain realistic. An improvement in growth velocity does not necessarily imply normalization of final height, complete correction of body disproportion, or prevention of all neurological, respiratory, or orthopedic complications. The long-term effects of treatment on final adult height, spinal canal development, foramen magnum dimensions, lower-limb alignment, pain, mobility, and quality of life remain under investigation.

Treatment response should not be assessed solely through standing height. Follow-up should include growth velocity, sitting height, arm span, body proportions, weight, head circumference in younger children, pubertal development, blood pressure, functional status, pain, mobility, and patient-reported outcomes. Monitoring should also identify adverse effects and changes in the natural history of the disorder. A major remaining uncertainty is whether sustained treatment modifies the natural history of clinically important complications rather than primarily increasing linear growth. Current evidence does not yet establish whether long-term vosoritide therapy reduces the incidence or severity of foramen magnum stenosis, spinal stenosis, lower-limb deformity, chronic pain, mobility impairment, or the need for orthopedic and neurosurgical procedures. Evidence regarding final adult height, long-term body proportionality, psychosocial outcomes, treatment burden, and health-related quality of life also remains incomplete. Prospective longitudinal studies and real-world registries will therefore be essential for determining whether early and sustained treatment translates into durable functional and structural benefit.

### 7.2. Integration with Multidisciplinary and Orthopedic Care

Vosoritide should be integrated into comprehensive multidisciplinary care rather than considered a stand-alone intervention. Children receiving treatment continue to require neurological, respiratory, orthopedic, otolaryngological, dental, rehabilitation, and psychosocial surveillance according to age and individual complication profile. Disease-modifying therapy does not eliminate the need for cranio-cervical imaging, sleep assessment, hearing evaluation, growth monitoring, or timely management of skeletal deformities.

The relationship between pharmacological treatment and orthopedic intervention remains incompletely defined. Increased longitudinal growth may influence the timing, planning, or expected outcomes of guided growth, corrective osteotomy, or limb-lengthening procedures. Conversely, orthopedic surgery may interrupt treatment temporarily or modify rehabilitation and monitoring requirements. Decisions should therefore be made collaboratively by pediatricians, endocrinologists, orthopedic surgeons, rehabilitation specialists, geneticists, and families [6,16].

When surgery is considered, treatment goals should extend beyond increased height and include pain reduction, improved alignment, mobility, self-care, independence, and participation. The potential benefits of combining vosoritide with orthopedic treatment should be weighed against surgical burden, complication risk, recovery time, and the preferences of the child and family.

### 7.3. Treatment Burden and Shared Decision-Making

Daily subcutaneous administration can represent a meaningful burden for children and families. Treatment requires sustained motivation, repeated monitoring, access to specialist services, and realistic understanding of expected outcomes. Injection-related anxiety, discomfort, treatment fatigue, school and travel logistics, and the duration of therapy should be discussed before initiation and revisited during follow-up.

Shared decision-making is therefore essential. Families should receive balanced information regarding the potential benefits, uncertainties, adverse effects, practical demands, and elective nature of treatment. Whenever developmentally appropriate, the child’s views should also be included. Decisions should reflect not only clinical eligibility, but also individual values, functional priorities, treatment goals, and willingness to continue long-term therapy.

The success of disease-modifying treatment should ultimately be evaluated through a multidimensional framework that includes growth, body proportions, complications, physical function, mobility, autonomy, psychosocial well-being, treatment burden, and quality of life. Height gain is an important outcome, but it should not be regarded as the sole measure of therapeutic benefit.

## 8. Quality of Life and Psychosocial Impact

### 8.1. Patients

Achondroplasia affects quality of life across the entire lifespan. Its impact goes beyond disproportionate short stature and medical complications, influencing mobility, independence, daily activities, and psychological well-being. Compared with the general population, individuals with achondroplasia tend to report lower quality of life, especially in physical domains, although mental health may also be affected [9,21]. In children and adolescents, the burden is particularly visible in physical and social functioning. Studies using QoLISSY and PedsQL show lower scores than in average-stature peers. Functional independence may develop more slowly, especially in younger children, who often need additional support with self-care, mobility, and daily tasks. This shows that achondroplasia in childhood is not only a disorder of reduced height, but also a condition with practical consequences for everyday life. Pain is one of the most consistent elements of disease burden. It may begin in adolescence, when more than half of patients report at least one painful site, most often involving the knees or shoulders. In adulthood, pain becomes one of the strongest contributors to reduced quality of life, appearing prominently in general health questionnaires and functional assessments [4,14,21]. It should therefore be regarded as a central clinical problem, not a secondary or occasional complaint.

In adults, the impact on health-related quality of life becomes broader. Many patients report limitations in mobility, difficulties with usual activities, persistent pain or discomfort, and symptoms of anxiety or depression [14]. Common everyday challenges include prolonged standing, climbing stairs, dressing, or reaching objects placed at height. These limitations show how the disease affects daily functioning beyond isolated orthopedic or neurological complications. Psychological well-being is also affected. Adults with achondroplasia often score lower than the general population across several SF-36 domains, especially pain, physical functioning, general health, and vitality. Brief Symptom Inventory assessments have also shown higher levels of psychological distress, including anxiety and phobic anxiety [4,9]. Emotional burden is therefore part of the lived experience of the condition, particularly in adulthood. The relationship between phenotype and quality of life is not simple. Short stature and disproportion clearly contribute to functional and psychosocial challenges, but final height alone does not fully explain quality of life. Some expert opinions suggest that final height below 140 cm may be associated with poorer physical HRQoL, while the relationship with mental well-being appears more variable and shaped by complications, adaptations, social environment, and individual coping strategies [16,22].

Treatment itself can also influence quality of life. Decisions should be individualized, and when possible, the child’s perspective should be included. Daily injections, for example, may be experienced as a meaningful burden by some patients and should be part of the benefit–risk discussion [15]. Quality of life is therefore not only an outcome to measure after treatment, but also a factor that should shape treatment decisions. Physical activity may have a protective role. Available data suggest that physically inactive adults tend to have poorer SF-36 scores and less favorable psychological profiles [4]. Although causality cannot be assumed, adapted physical activity may support both physical function and psychosocial well-being. The patient burden of achondroplasia is cumulative and changes with age. It affects skeletal growth, but also mobility, independence, self-perception, mental health, and social participation. Routine follow-up should therefore include pain, function, mobility, and quality of life, alongside surveillance for medical complications (Table 5) [16,24].

Table 5 demonstrates that the impact of achondroplasia cannot be adequately represented by stature alone. During childhood and adolescence, reduced independence, delayed acquisition of some motor and self-care skills, participation restrictions, pain, and social experiences may influence quality of life. In adulthood, chronic pain, mobility limitations, fatigue, environmental barriers, and difficulties with daily activities become increasingly prominent. Psychological outcomes show substantial interindividual variability and may be influenced by coping, family support, stigma, accessibility, and the social environment. Because available studies use different instruments and frequently include small or cross-sectional samples, apparent age-related differences should not be interpreted as direct longitudinal changes within the same individuals [4,9,14,15,16,21,22,24].

### 8.2. Families

The impact of achondroplasia extends beyond the patient and directly involves the family. In most cases, the child is born to average-stature, unaffected parents, since more than 80% of cases arise de novo. For many families, the diagnosis is unexpected and brings an immediate need to understand complex medical information, uncertain prognosis, and long-term surveillance [21]. Counseling is particularly important during the prenatal and early postnatal periods. Families need clear information about possible complications in the first year of life, birth planning, postnatal follow-up, and warning signs that require urgent care. They also need to understand the care pathway itself, including early neurological, respiratory, and orthopedic monitoring [15]. The practical burden of care is substantial. Access to specialized services may be limited by distance from expert centers, transport difficulties, long waiting times, shortage of trained specialists, or insurance and reimbursement barriers. Families therefore manage not only the medical aspects of the condition, but also a continuous organizational effort that can interfere with work, routines, and family life [1,15].

Family participation in treatment decisions is essential. This is especially clear in high-risk situations such as decompression for foramen magnum stenosis, where parents must understand the rationale, risks, and expected benefits of surgery. Shared decision-making can improve trust, satisfaction, and cooperation, supporting a model of care that is genuinely family-centered [12]. The same applies to pharmacological treatment. Before starting vosoritide, families should receive balanced information about expected benefits, limitations, adverse effects, the elective nature of therapy, and the practical burden of daily injections. Informed consent should be a real process, not a formality. Parents need to understand both disease-related risks and treatment-related demands, and decisions should reflect the values and priorities of the family [24]. The psychosocial experience of parents remains poorly studied. One review identified 1549 records, yet only two studies met inclusion criteria, showing how limited the evidence base still is. Even so, the available data are consistent: parents may experience persistent stress, social isolation, changes in family dynamics, occupational and financial strain, and exhaustion related to repeated interactions with the healthcare system. Achondroplasia also differs from many other chronic childhood conditions because it is visible. Parents may worry not only about medical complications, but also about stigma, bullying, physical development, and the child’s future independence. This gives the parental experience a strongly social and anticipatory dimension [1].

Another important issue is the gap between parent and child perspectives. In quality-of-life studies, parents often rate the impact of achondroplasia as more severe than children do themselves [14,16]. This difference does not invalidate parental reports, but it means they should be interpreted carefully. It also shows that supporting parents is not secondary, since parental well-being may influence the child’s own adjustment and quality of life. Psychological support becomes particularly important when complex surgical decisions are discussed. Families considering repeated orthopedic procedures or limb lengthening may face ethical uncertainty, fear of complications, emotional pressure, and difficulty balancing functional benefits against physical and psychological burden. The lack of universal consensus for some of these decisions makes careful communication and ongoing support even more important [16]. The family should therefore be treated as part of the unit of care, not simply as the patient’s background. Relatives, friends, and advocacy groups can help families adapt and develop coping strategies, but professional support remains important. Routine psychosocial assessment, anticipatory counseling, and clear referral pathways to psychological and community resources should be part of modern achondroplasia care [1,21].

### 8.3. Social Integration

Social integration in achondroplasia is shaped by several overlapping factors: functional limitations, inaccessible environments, and the psychological effect of visible difference. Participation therefore depends not only on medical status, but also on practical adaptations that allow independence in daily life. Modifications at home, school, work, and in the wider community, together with appropriate assistive devices, can make a substantial difference [9,21]. In childhood, school is one of the most important settings for social participation. The aim is not simply attendance, but participation with as much independence as possible. Useful accommodations may include steps or supports in the classroom, adapted desks and chairs, and accessible bathroom arrangements. When necessary, these measures can be formalized through an individualized educational plan to ensure continuity throughout the school year. How achondroplasia is discussed at school also matters. Families and clinicians may need to think carefully about how dwarfism is explained to classmates, what terminology is used, and how bullying can be prevented. Open communication and age-appropriate language may help the child respond to questions from peers with more confidence. Peer groups for people with short stature can also be valuable, and in some cases psychological support for the child or family may be appropriate [15]. These issues do not disappear in adulthood. Psychosocial health should be reviewed routinely, with referral to psychological or psychosocial support when needed [9]. The need for adaptations may also change over time. Home, workplace, transport, and community accessibility should therefore be revisited during follow-up, because each of these can influence independence and participation. Mobility illustrates this clearly. For many adults with short stature, access to a car is central to independence [9]. This example shows that social inclusion is not only about reducing stigma. It also depends on whether people can physically access education, work, healthcare, leisure activities, and social relationships. Quality-of-life data support the importance of social participation. In children and adolescents with achondroplasia, social functioning is consistently among the more affected domains in QoLISSY and PedsQL assessments [14]. This likely reflects a combination of functional limitations, reduced autonomy, environmental barriers, and challenges related to self-image or peer interaction.

In adults, social difficulties may persist in different forms. Studies describe restricted participation, difficulties in interpersonal relationships, negative stereotypes related to short stature and competence, and reduced material well-being. Social functioning has also been strongly associated with psychological health measures, suggesting a two-way relationship: reduced participation may worsen emotional distress, while psychological distress may further limit engagement [4]. Stigma is an important part of this experience. People with achondroplasia and their families may face unwanted attention, intrusive questions, or persistent stereotypes. Some families may withdraw from social situations or struggle to relate to others without similar experiences. Formal and informal support can be protective, while support groups and education about the condition may strengthen resilience and provide a greater sense of control [1]. Social integration should therefore be treated as a therapeutic goal, not a secondary issue. Managing medical complications is not enough if patients remain limited by inaccessible environments, low autonomy, or social exclusion. Effective care should combine practical accommodations, psychological support, education, and anti-stigma strategies to support meaningful participation in school, work, family life, and the wider community [9,15,21].

## 9. Discussion and Future Perspectives

A further challenge is the gap between recommended care and real-world implementation. Consensus documents generally favor coordinated multidisciplinary surveillance and access to clinicians experienced in achondroplasia; however, such models may not be available in all regions. Adult services are particularly fragmented, and geographical distance, reimbursement limitations, shortages of specialist expertise, and differences between healthcare systems can restrict access to recommended surveillance and treatment. Shared-care models linking specialized centers with local clinicians, supported where appropriate by telemedicine, may provide a pragmatic strategy for improving continuity without assuming that every patient can receive all care within a single expert center.

Important uncertainties also remain regarding the optimal intensity and timing of surveillance, the long-term consequences of disease-modifying treatment, and the extent to which recommendations developed in highly specialized centers can be generalized to other healthcare settings. Differences in patient age, phenotype severity, study design, outcome definitions, follow-up duration, and access to specialist services complicate direct comparison across studies. These limitations are particularly relevant for psychosocial outcomes, adult healthcare, transition, and quality-of-life research, where available samples are often small and cross-sectional.

The available literature provides a relatively consistent description of the major clinical manifestations of achondroplasia, but the strength of evidence supporting specific surveillance and management strategies varies considerably across domains. Neurological and respiratory surveillance during infancy is supported by accumulated clinical experience, observational evidence, and international consensus, whereas evidence concerning adult care, structured transition programs, psychosocial interventions, caregiver support, and long-term functional outcomes remains substantially more limited. Consequently, several current recommendations represent expert consensus rather than conclusions derived from prospective comparative studies. This distinction is important when translating published guidance into individualized clinical practice.

Future progress in achondroplasia care will depend not only on the development of new pharmacological therapies, but also on improving the organization, continuity, and patient-centered nature of lifelong clinical management. One of the most important priorities is the establishment of standardized surveillance pathways that can be applied consistently across specialized and non-specialized healthcare settings. Although several clinical recommendations are available, differences remain regarding imaging schedules, sleep assessment, orthopedic monitoring, transition to adult care, and long-term follow-up. International registries and prospective longitudinal studies will be essential for defining the natural history of achondroplasia more precisely and for evaluating the long-term effects of disease-modifying treatment. Real-world evidence is particularly important for infants and young children who begin therapy early, because clinical trial populations and follow-up periods may not capture the complete spectrum of outcomes. Long-term studies should evaluate not only height and growth velocity, but also foramen magnum dimensions, spinal canal development, lower-limb alignment, pain, mobility, functional independence, cardiovascular health, hearing, and quality of life.

The potential influence of vosoritide and future disease-modifying therapies on structural complications remains uncertain. It is not yet known whether early treatment can reduce the incidence or severity of foramen magnum stenosis, spinal stenosis, lower-limb deformity, or the need for orthopedic surgery. Prospective imaging and clinical studies are therefore needed to determine whether pharmacological treatment modifies skeletal development beyond longitudinal growth.

Another major area of uncertainty concerns the integration of disease-modifying therapy with orthopedic management. Pharmacological treatment may alter growth patterns and consequently influence the optimal timing of guided growth, corrective osteotomy, or limb lengthening. At present, standardized protocols for combining these interventions are lacking. Future recommendations should be based on prospective multidisciplinary data and should include outcomes valued by patients, such as mobility, pain reduction, self-care, independence, and participation.

Transition from pediatric to adult care requires particular attention. Many adults with achondroplasia experience fragmented follow-up despite an increasing burden of spinal stenosis, chronic pain, mobility limitations, obesity, sleep-disordered breathing, hearing impairment, and cardiovascular risk. Future care models should establish clear referral pathways, define the role of primary care, improve access to experienced specialists, and provide adults with practical information regarding warning symptoms and recommended surveillance.

Patient-reported outcomes should become a central component of both clinical care and research. Height alone does not adequately represent the burden of achondroplasia or the value of treatment. Validated tools are needed to assess pain, fatigue, mobility, self-care, social participation, emotional well-being, treatment burden, and family impact across different age groups. Patient and caregiver perspectives should also be incorporated into the development of clinical guidelines, research priorities, and regulatory outcomes.

Access to specialized care and disease-modifying therapy remains unequal. Geographical distance, cost, reimbursement policies, limited specialist expertise, and differences between healthcare systems may restrict access to diagnosis, surveillance, and treatment. Telemedicine, regional networks, shared-care models, and standardized digital monitoring tools may improve continuity and reduce some of these barriers.

The future of achondroplasia management is therefore likely to involve individualized, multidisciplinary, and lifespan-based care. Pharmacological treatment, orthopedic intervention, rehabilitation, psychosocial support, environmental adaptation, and patient education should be integrated according to age, phenotype, complications, functional priorities, and personal preferences. The ultimate goal should not be limited to increasing height, but should include prevention of complications, preservation of function, improved autonomy, meaningful participation, and lifelong well-being.

Key research priorities therefore include long-term evaluation of disease-modifying therapy using functional and quality-of-life outcomes; prospective assessment of its effects on major structural complications; validation of transition programs; development and evaluation of sustainable adult-care models; and longitudinal assessment of caregiver and patient-reported outcomes. These questions require multicenter prospective cohorts, registries, and real-world studies with standardized outcome definitions.

## 10. Limitations of the Review

This review has several limitations. Although the literature search incorporated multiple bibliographic databases and supplementary citation searching, its narrative design does not provide the exhaustive retrieval guarantees of a systematic review, and selection and interpretation bias remain possible. No formal study-level risk-of-bias or certainty-of-evidence assessment was performed. The available evidence is heterogeneous with respect to age, study design, sample size, outcome definitions, follow-up duration, and healthcare context. Several domains, particularly caregiver burden, adaptive functioning, transition to adult healthcare, healthcare inequalities, adult models of care, and long-term patient-reported outcomes, are supported predominantly by small, cross-sectional, descriptive, or consensus-based evidence. Accordingly, some clinical recommendations discussed in this review reflect expert consensus rather than high-certainty empirical evidence. Finally, findings and recommendations may have limited generalizability across healthcare systems with substantially different access to specialized services and disease-modifying therapies.

## 11. Conclusions

Achondroplasia is a lifelong multisystem skeletal disorder whose clinical consequences extend far beyond disproportionate short stature. Neurological, respiratory, orthopedic, otolaryngological, cardiovascular, metabolic, oral, functional, and psychosocial complications may appear at different stages of life and require age-specific surveillance and coordinated multidisciplinary management.

During infancy and early childhood, care should prioritize the detection of foramen magnum stenosis, cervicomedullary compression, sleep-disordered breathing, hypotonia, and developmental concerns. Later childhood and adolescence require increasing attention to orthopedic deformities, pain, hearing, mobility, physical independence, and psychosocial adaptation. In adulthood, spinal stenosis, degenerative musculoskeletal disease, chronic pain, reduced mobility, cardiometabolic risk, and continuity of specialist care become major priorities.

Vosoritide has expanded the therapeutic options available for children with achondroplasia, but disease-modifying treatment does not replace established surveillance, rehabilitation, orthopedic care, psychosocial support, or environmental adaptation. Its long-term value should be assessed through outcomes that extend beyond growth velocity and include skeletal complications, physical function, autonomy, treatment burden, participation, and quality of life.

Optimal management of achondroplasia should follow a lifespan-based strategy integrating medical surveillance with developmental functioning, rehabilitation, psychosocial care, environmental adaptation, and family support. Priorities should evolve with age, from prevention of neurological and respiratory complications in infancy to functional independence, educational and social participation, transition preparation, chronic-pain management, adult mobility, and long-term psychosocial well-being. Disease-modifying therapy should be incorporated into this broader framework and evaluated using outcomes extending beyond growth velocity to include function, structural complications, autonomy, treatment burden, participation, and quality of life.

At the health-policy level, priorities should include standardized national or regional surveillance pathways, multidisciplinary referral networks, structured pediatric-to-adult transition programs, equitable access to specialist and disease-modifying care, and improved availability of rehabilitation, psychological, educational, and family-support services. Rare-disease registries, prospective real-world data collection, telemedicine, and shared-care networks may further improve continuity of care and support evidence-based planning across the lifespan.

## Figures and Tables

**Table 1:** Predominant clinical manifestations across different life stages in achondroplasia. Synthesized from published clinical recommendations and review data regarding age-related manifestations and complications in achondroplasia.

Life Stage	Common Manifestations/Priorities
Prenatal period	Femoral shortening, macrocephaly, rhizomelic disproportion, prenatal ultrasound suspicion
Neonatal period/infancy	Foramen magnum stenosis, hypotonia, sleep-disordered breathing, feeding difficulties, hydrocephalus surveillance
Early childhood	Delayed motor milestones, thoracolumbar kyphosis, recurrent otitis media, hearing impairment, obstructive sleep apnea
School age	Genu varum, spinal alignment abnormalities, speech/language issues, psychosocial adaptation, dental/orthodontic problems
Adolescence	Chronic pain, obesity risk, progressive limb deformity, reduced exercise tolerance, psychosocial burden
Adulthood	Spinal stenosis, chronic pain, cardiovascular risk, reduced mobility, osteoarthritis, functional limitation

Source: Authors’ elaboration based on references [3,9,15,16,21,24].

**Table 2:** Major complications of achondroplasia: mechanisms, clinical features and management. Synthesized from published clinical recommendations and review data on foramen magnum stenosis, spinal stenosis, sleep-disordered breathing, ENT complications, cardiovascular risk, and oral manifestations in achondroplasia.

Complication	Pathophysiology	Key Clinical Features	Screening/Diagnosis	Management
Foramen magnum stenosis	Impaired skull base growth, premature synchondrosis closure leading to cervicomedullary compression	Hypotonia, apnea, developmental delay, feeding difficulties	Neuro exam, polysomnography, MRI (early infancy)	Surgical decompression when indicated
Spinal stenosis	Congenital narrow canalwith degenerative changes in discs, ligaments, facets	Back pain, neurogenic claudication, gait limitation, weakness	MRI spine, neurological exam	Decompression surgery with or without fusion
Sleep apnea (OSA/CSA)	Airway obstruction due to midface hypoplasia and adenotonsillar hypertrophy; brainstem compression may also contribute	Snoring, apnea, daytime sleepiness, feeding issues (infants)	Polysomnography	Adenotonsillectomy, CPAP, neurosurgery if central cause
ENT complications	Eustachian tube dysfunction, craniofacial anatomy	Recurrent otitis media, hearing loss, speech delay	Audiology, ENT assessment	Tympanostomy tubes, adenotonsillectomy
Cardiovascular risk	Obesity, reduced activity, sleep apnea, metabolic factors	Hypertension, reduced exercise tolerance, early CV events	BP monitoring, metabolic assessment	Lifestyle measures, risk factor control
Oral and dental manifestations	Midface hypoplasia, maxillary constriction, altered craniofacial growth	Class III malocclusion, posterior crossbite, macroglossia, open bite, speech difficulties, oral breathing	Dental and orthodontic assessment, panoramic imaging when indicated	Early orthodontic evaluation, preventive dental care, adapted airway and positioning precautions during treatment

Source: Authors’ elaboration based on references [3,8,9,12,15,16,17,20,21,24].

**Table 3:** Diagnostic approach to achondroplasia: clinical, imaging, and genetic components. Synthesized from published clinical recommendations and review data on clinical diagnosis, imaging assessment, prenatal detection and molecular confirmation of achondroplasia.

Diagnostic Domain	Key Findings	Typical Timing	Clinical Role
Clinical evaluation	Macrocephaly, frontal bossing, midface hypoplasia, rhizomelic limb shortening, trident hand	Neonatal period; may become more evident with growth	Initial suspicion and phenotypic recognition; early identification of complications
Prenatal assessment	Femoral shortening, macrocephaly, facial profile changes on ultrasound	Usually after 24 weeks of gestation	Raises suspicion; guides need for further testing
Conventional radiography	Narrow interpedicular distance (lumbar), square iliac bones, metaphyseal flaring, short femoral neck	Postnatal (neonatal period and early childhood)	Confirms skeletal phenotype; supports diagnosis
MRI (cranio-cervical and spine)	Foramen magnum narrowing, cervicomedullary compression, spinal canal stenosis	Infancy (routine at 3–6 months) and when symptomatic	Detects life-threatening complications; guides neurosurgical decisions
Genetic testing (targeted FGFR3)	Identification of c.1138G>A or c.1138G>C (p.Gly380Arg)	Any stage (prenatal or postnatal)	Confirms diagnosis; required for treatment decisions (e.g., vosoritide)
Extended genetic testing	Rare FGFR3 variants or alternative diagnoses	When targeted testing is negative but suspicion persists	Clarifies atypical cases and differential diagnosis
Prenatal molecular testing	CVS, amniocentesis, or single-gene NIPT (selected cases)	Prenatal	Confirms diagnosis before birth; supports counseling and planning

Source: Authors’ elaboration based on references [7,12,15,21,24,29].

**Table 4:** Core Components of Transition from Pediatric to Adult Healthcare in Achondroplasia.

Domain	Actions Before Transfer	Information to Be Transferred	Adult Follow-Up Priorities
Neurological and spinal care	Review symptoms, examination and previous imaging	Cranio-cervical and spinal MRI, decompression history, neurological deficits	Spinal stenosis, neurological red flags, mobility decline
Respiratory and sleep care	Reassess sleep symptoms and respiratory support	Polysomnography, OSA/CSA history, CPAP and ENT procedures	Persistent or recurrent sleep-disordered breathing
Orthopedic care	Review deformities, gait and surgical history	Limb alignment, spinal deformity, osteotomies and rehabilitation	Degenerative disease, pain and need for further intervention
Pain and mobility	Document pain, walking ability and assistive devices	Functional assessments, analgesic use, mobility aids	Chronic pain, function, participation and independence
Cardiometabolic health	Establish baseline adult risk profile	Blood pressure, weight trends, activity level and metabolic results	Hypertension, obesity and cardiovascular risk
Hearing and communication	Perform audiological reassessment when indicated	Hearing loss, hearing aids, tympanostomy and ENT history	Continued hearing and communication surveillance
Psychosocial and adaptive functioning	Assess autonomy, emotional health and support needs	Educational needs, employment goals, mental health history	Independence, employment, accessibility and well-being
Genetic and reproductive care	Provide age-appropriate genetic counseling	Molecular diagnosis, inheritance information and family history	Reproductive counseling and pregnancy planning
Care coordination	Identify adult clinicians and referral pathways	Complete transition summary and contact information	Continuity of multidisciplinary care

Source: Authors’ elaboration based on references [21,32,33,34,35].

**Table 5:** Impact of achondroplasia on patient quality of life across domains. Synthesized from published studies and expert reviews on health-related quality of life, pain, physical functioning, psychosocial burden and physical activity in children, adolescents and adults with achondroplasia.

Domain	Children/Adolescents	Adults	Key Contributing Factors
Physical functioning	Delayed motor development, reduced independence	Reduced mobility, fatigue, limitations in daily activities	Skeletal deformities, short stature, pain
Pain	May begin in adolescence	Chronic, often multisite	Joint stress, spinal pathology, biomechanics
Social functioning	Challenges in participation, peer interaction	Reduced participation, environmental barriers	Accessibility issues, stigma
Psychological well-being	Variable, influenced by family and environment	Anxiety, distress, reduced vitality	Chronic disease burden, social factors
Daily activities	Need for assistance in self-care (early ages)	Difficulty with routine tasks (stairs, reaching, dressing)	Functional limitations
Quality of life scores	Lower QoLISSY/PedsQL scores	Lower SF-36 scores	Multidimensional disease burden
Modifying factors	Family support, early interventions	Physical activity, coping strategies	Social environment, adaptation

Source: Authors’ elaboration based on references [4,9,14,15,16,21,22,24].

## Data Availability

No new data were created or analyzed in this study. Data sharing is not applicable to this article.
